# Proteins from Agri-Food Industrial Biowastes or Co-Products and Their Applications as Green Materials

**DOI:** 10.3390/foods10050981

**Published:** 2021-04-29

**Authors:** Estefanía Álvarez-Castillo, Manuel Felix, Carlos Bengoechea, Antonio Guerrero

**Affiliations:** Departamento de Ingeniería Química, Escuela Politécnica Superior, 41011 Sevilla, Spain; malvarez43@us.es (E.Á.-C.); mfelix@us.es (M.F.); aguerrero@us.es (A.G.)

**Keywords:** bioplastic, protein, biowaste, valorization

## Abstract

A great amount of biowastes, comprising byproducts and biomass wastes, is originated yearly from the agri-food industry. These biowastes are commonly rich in proteins and polysaccharides and are mainly discarded or used for animal feeding. As regulations aim to shift from a fossil-based to a bio-based circular economy model, biowastes are also being employed for producing bio-based materials. This may involve their use in high-value applications and therefore a remarkable revalorization of those resources. The present review summarizes the main sources of protein from biowastes and co-products of the agri-food industry (i.e., wheat gluten, potato, zein, soy, rapeseed, sunflower, protein, casein, whey, blood, gelatin, collagen, keratin, and algae protein concentrates), assessing the bioplastic application (i.e., food packaging and coating, controlled release of active agents, absorbent and superabsorbent materials, agriculture, and scaffolds) for which they have been more extensively produced. The most common wet and dry processes to produce protein-based materials are also described (i.e., compression molding, injection molding, extrusion, 3D-printing, casting, and electrospinning), as well as the main characterization techniques (i.e., mechanical and rheological properties, tensile strength tests, rheological tests, thermal characterization, and optical properties). In this sense, the strategy of producing materials from biowastes to be used in agricultural applications, which converge with the zero-waste approach, seems to be remarkably attractive from a sustainability prospect (including environmental, economic, and social angles). This approach allows envisioning a reduction of some of the impacts along the product life cycle, contributing to tackling the transition toward a circular economy.

## 1. Introduction

The accumulation of plastic wastes is a globally recognized problem that involves an extremely negative impact on the environment [1]. The exceptionally low biodegradability of fossil-based plastics, together with the massive production scale associated with the plastic market over the past 60 years, has generated a huge accumulation of plastics in landfills and the oceans [2]. To illustrate the magnitude of the problem, considering that almost 400 Mt of plastic waste is generated every year [3], there is currently more than 1 ton of plastic/person alive in the world. However, in spite of the recent efforts made in this field to shift from a fossil-based to a bio-based circular economy model, only 20% of plastic is collected for recycling, of which only 3% is reused [4,5]. The rest is incinerated, landfilled, or disposed of into nature, an large part of which is ending up in the oceans [6]. In this sense, European Union Directive (EU) 2019/904 aims to prevent and reduce the impact of certain single-use plastic products on the environment, especially the marine environment, and on human health. Consequently, the future of the plastics industry needs to be driven by sustainability issues, where the bioplastic sector is a crucial building block for a circular economy scenario [7,8].

The most accepted definition of the term bioplastic, which has been controversial among plastic industrial associations and environmental organization, is given by European Bioplastics [5]. According to this association, any plastic material can be denoted as a bioplastic if it is either bio-based, biodegradable, or displays both properties. Consequently, bioplastics embrace a whole family of materials with different properties and applications, ranging from biodegradable fossil-based polymers, such as poly(butylene adipate-co-terephthalate) (PBAT) or polycaprolactone (PCL), to non-biodegradable bio-based polymers, such as bio-based polyolefins (e.g., bioPE and bioPP) or polyesters (e.g., bioPET) [9]. However, the ecofriendliest bioplastic group is formed by biodegradable and bio-based polymers. This group comprises biodegradable aliphatic polyesters produced by fermentation of biomass, including polylactates (PLA), polyglycolates (PGA), polyhydroxy butyrate (PHB), polyhydroxy valerate (PHV), etc., and polymers extracted from renewable sources, also known as agropolymers, which include polysaccharide-based polymers (e.g., starch, cellulose, and cellulose derivatives) and protein-based polymers that can be extracted from animal or plant sources [10,11]. Currently, a big amount of the food produced worldwide (~30%) is discarded by the agri-food industry, being considered as byproducts or wastes [12]. These food biowastes could be reused as raw materials for the emerging bioplastics sector since their proteins, carbohydrates, lipids, and other compounds can be used for this application [13]

Agropolymers are considered the most ecoefficient bioplastic source in terms of the ratio between the added value of their potential applications and the environmental impacts associated with them [14]. They consist of a carbon backbone with different side groups that can form inter-/intra-molecular H-bonds. It is precisely the ability to temporarily disrupt these H-bonds and cause flow into new material shapes that allows forming plastic materials by conventional polymer processing techniques (e.g., casting, thermoforming, compression molding, extrusion, and injection molding) [15]. However, despite the unquestionable importance of bioplastics for enabling a more sustainable circular economy [16], they only cover approximately 1% of the global plastic market, accounting for 2.11 Mt in 2020 [5]. About 60% of the bioplastic market corresponds to biodegradable polymers and 20% to agropolymers (over 420 kt). Among them, starch and cellulose are abundant and low-priced raw materials [6]. Unfortunately, they typically require complex processing before they can be properly used as bioplastics. These processes, including fermentation or functionalization, typically increase their costs and, as a result, reduce their efficiency in the replacement of conventional plastics.

In contrast, an emerging ecofriendly and cost-efficient alternative to plastics is based on the use of protein which can be easily processed for many applications [17,18]. Moreover, protein may be inexpensively extracted from many sources that are also abundant in nature. Interestingly, global food biowastes represent about 1300 Mt/year, according to the Food and Agriculture Organization (FAO) of the United Nations [19]. This biomass may be regarded as a potential source that can be used in the protein-based bioplastic sector, competing with other uses (e.g., biofuel). However, some problems related to the collection of food biowastes, due to their extremely wide dispersion, still impose a barrier to their efficient application at large scales [20]. Other more interesting alternatives are currently being considered for the valorization of proteins, such as the use of agri-food co-products from the starch, oil, or biofuel industries; the extraction from industrial biowastes such as blood, bones, feathers, wool, hair, nails, etc., from poultry or cattle slaughterhouses; or microalgae from sewage plants [20,21]. However, the commercial use of protein-based bioplastics in 2020 is still residual (with an output lower than 30 kt) as compared to other agropolymers, particularly starch which accounts for almost 400 kt in 2020 [5]. Some authors have indicated that plastics production from proteins is economically feasible, reducing the wastes associated with industrial products [22].

As for the portfolio of bioplastic applications, food packaging remains the widest segment of the whole bioplastic family, with an output of almost 1 Mt in 2020, representing nearly half of the total bioplastics market. The other half is largely diversified finding applications as consumer goods, or in the textile, agriculture, automotive, and construction field, among others [5]. In particular, protein-based bioplastics may also accomplish some of those applications, mainly in the fields of food packaging and plastics for agriculture. Moreover, they may also be used in more specific applications, such as in the development of absorbent and superabsorbent materials, in the controlled release of active agents (e.g., drugs, antimicrobial agents, nutrients, etc.), or biomedical applications (e.g., as scaffolds for tissue engineering) [23]. Therefore, despite the many advantages associated with the use of protein as bioplastics for a wide variety of applications, its high potential for the replacement of conventional plastics has not yet been sufficiently explored [20].

This review is focused on the potentials of protein co-products of the agri-food industry or protein fractions extracted from agri-food industrial biowastes, as well as their applications as substitutes for conventional non-biodegradable fossil-based plastics. The characteristic of these protein-based materials must be analyzed to assess the functionality required for each application. These properties can be typically divided into mechanical properties, thermal properties, and optical properties, correlating them to the microscopic (even molecular) structure of the materials [24].

## 2. Proteins from Industrial Biowastes and Co-Products

Every year, around one third of all food produced worldwide is either lost or wasted [25]. In Europe, that amount is reduced to one fifth, being 19% obtained from food processing and 11% from primary production [12]. Food biowaste is mainly composed of carbohydrates, proteins, lipids, and other compounds with great potential for high-value applications [13]. In this section, the main protein-rich biowastes and co-products from the agri-food industry that have been used in the development of plastic materials are presented. It should be highlighted that depending on the application pursued, proteins should be previously extracted and/or concentrated from the biowaste. Extraction can be carried out either through dry (e.g., air classification) or nondry (e.g., chemical treatment) conditions. Among the concentration procedures for obtaining protein concentrates or isolates are isoelectric precipitation or ultrafiltration. These preparation techniques are outside of the scope of this study, and readers interested in their description are referred to a recent review on this topic [26].

### 2.1. Co-Products from Starch

Wheat gluten (known as vital gluten, containing 75–80% protein) and corn gluten (typically containing 55–70% protein) are produced industrially as a co-product either from starch or bioethanol industrial plants [27,28,29]. The most abundant amino acid residues present in wheat gluten are glutamate and glutamine (31.9%) and proline (14.1%) [30]. Corn gluten is abundant in methionine and cysteine but is very low in lysine and tryptophan [31]. Wheat gluten is mainly used in bakery products, while corn gluten is mainly used as animal feed. Nonfood applications for gluten have been pursued (e.g., thermoplastic materials) [32,33]. In this sense, most wheat gluten-based plastics have been processed through casting or extrusion [33,34,35,36], leading to insoluble films, plastics, and adhesives with good barrier properties for oxygen and carbon dioxide [37,38]. Corn gluten has not been studied as profusely as wheat gluten in the field of bio-based materials. However, some studies have reported its potential to form glassy dense material of high thermoplasticity [29,39].

Potato proteins (i.e., patatin, protease inhibitors, and different high-molecular-weight proteins) can be obtained from potato-based starch production, as well as from peels and damaged potatoes. Although the protein content of fresh potatoes is low (2%), a protein isolate (90%) can be obtained from the wastewater generated during their processing through alkaline precipitation [40,41]. Potato protein is rich in hydrophobic amino acid residues with branched (isoleucine (3.1%), leucine (6.7%), and valine (3.7%)) and aromatic (phenylalanine (4.2%) and tyrosine (3.8%)) side chains [42,43]. They also possess a lysine content (~6%) higher than the average found in most plant-based proteins [43]. Potato proteins have been used as food additives or for bioplastic production. Thus, films or sheets from thermoforming or compression molding have been produced from potato protein flours mold, leading to bioplastics with adequate mechanical properties, sometimes reinforced with some animal proteins, such as gelatin [44,45]. Films obtained by casting have also proven to show significant barrier properties, highlighting its potential in the packaging industry [46].

### 2.2. Co-Products from Bioethanol

In addition to the abovementioned wheat gluten, zein can be also obtained as a co-product of the bioethanol industry. Zein is the alcohol-soluble protein of corn, and it is a prolamin predominantly present in the endosperm [47]. It possesses a negligible content in essential amino acids, such as lysine and tryptophan, which, together with its poor solubility in water, limit its use for human consumption [47]. Zein may be obtained as a byproduct from the production of ethanol, starch, or oil from corn, containing a high amount of glutamic acid (21–26%) and hydrophobic amino acids, like proline (10.5%) or leucine (21.1%) [47,48]. It has been mainly employed as a coating agent due to its ability to form films with water vapor barrier properties [49,50]. Zein-based films have also been produced through extruders provided with slit dies, where additives like oleic acid can be used to enhance elongation [51,52,53]. Furthermore, zein may be used as plasticizer in injection molded starch-based plastics [54].

### 2.3. Co-Products from Seed Oil

Soy oil is extracted from soybean (38–45% protein content) producing a protein-rich meal as a byproduct, which is, for the most part, discarded as industrial biowaste or used for feeding animals [55,56,57]. Soy proteins, mostly globulins, are rich in polar amino acid residues, such as glutamic acid (12.4%), also containing a considerable amount of lysine (3.4%) [43]. Soy protein-based bioplastics have been processed through several techniques, like casting, compression, or injection molding, resulting in materials with adequate mechanical properties but low water resistance [58,59,60,61]. It has been acylated successfully to further enhance its hydrophilic character, which may be well used in superabsorbent or horticulture applications [62,63].

The processing of rapeseed to obtain oil results in the production of a press cake with a high protein content (35%) [64], which cannot be used as a food ingredient due to the presence of antinutritional compounds (e.g., glucosinolates) [65]. Rapeseed and canola have been sometimes used interchangeably, although canola should be strictly employed for cultivars that have been genetically improved and contain a lower content of antinutritional compounds [66]. Main rapeseed proteins are globulin cruciferin (60%) and albumin napin (20%) [67,68], containing an important amount of glutamine/glutamate and aspartic acid/aspartate residues (18.14% and 7.25%, respectively) [69]. The protein-rich biowaste obtained in the manufacture of the oil is mainly used for low-value applications [70,71], although some research about high-value applications has been pursued. Plastic materials have been obtained from canola mostly through casting [72,73,74] or from rapeseed by compression molding or injection molding [75,76].

Sunflower cake after extracting sunflower oil has been used for the development of protein-based bioplastics [77,78,79]. The protein content of the cake after oil extraction is high (~35%); however, the lignocellulose content is also high (~40%) [77]. Within the protein fraction, globulins are the most abundant (~58%), followed by albumins (~20%), glutelins (~14%), and prolamins (~3%). Because of its high protein content, it has mostly used for animal feed. A protein extract can be obtained at alkali pH with a high content of globulin and albumin [79]. Films have been obtained from sunflower through casting or extrusion [79,80].

### 2.4. Wastes from Animal Farming

Both casein and β-lactoglobulin are milk proteins extensively used by the food industry. However, they are also noticeably present in the wastewater from dairy factory plants (casein) or in the whey from cheese production (β-lactoglobulin), which can be revalorized as a source of protein for the development of bioplastics [81]. Both sources are rich in glutamic acid (13.9% and 15.5%, respectively) and lysine (4.6% and 7.1%, respectively) [43]. Materials obtained from whey are similar to those prepared from caseins, characterized by transparency and flexibility and a water resistance that can be increased by crosslinking [82,83].

Blood (18% protein content) represents up to 4% of the animal weight and, and only 30% is used by the food industry, which would imply that over 3000 ML were discarded into municipal sewers and landfills in 2016 [84,85,86]. Plasma obtained after a centrifugation/drying process possesses a protein content that lies around 70% [87], which consists mainly of albumin (50–60%) and globulins (40–50%) [88]. The protein content is highly dependent on the animal species, being higher for bovine and porcine blood (~19%) and plasma (~6.9%) compared to poultry (~13 and 3.5%, respectively). Lysine (~7%), aspartic (~9.1%), and glutamic (~9.7%) acid contents are high for all those species [89,90]. Blood and plasma may be used for nonedible applications, such as packaging [91]. Thus, blood meal has been successfully extruded and injection molded [92,93,94], while more recently the plasma fraction has proven its potential as the basis of superabsorbent materials [95,96,97].

Around one third of the fish caught globally is used to produce protein-rich marine byproducts for animal feeding. For instance, a fish meal containing 59.0–68.5% of protein may be obtained [98]. Moreover, during fish processing, around 20–80% of waste, depending on the level of processing and type of fish, is generated which can also be used as fish meal [99]. Fish biowaste can also be used for production of proteins, oil, amino acids, minerals, enzymes, bioactive peptides, collagen, and gelatin. Most of the fishmeal is consumed by the aquaculture industry, but it could be employed for the production of green materials, through compression or extrusion [99,100].

Collagen represents 30% of the animal protein content and may be obtained from different byproducts of the meat industry, mostly pig skin (46%), bovine hide (29%), and pork and cattle bones (23%) [101]. One third of collagen is glycine, which is also rich in proline and hydroxyproline residues (~23% of the overall amino acid composition) [102,103]. Gelatin is produced when collagen is cooked or denatured by heat, being relatively cheap and abundant [104,105,106]. Its excellent ability to form films for both food and biomedical applications has facilitated its processing through casting, extrusion or electrospinning, displaying flexibility, good moisture and oxygen barrier properties, and excellent biodegradability [83,107,108].

Keratin comprises a mixture of high-molecular-weight fibrous proteins whose properties are greatly influenced by the methodology (chemical, enzymatic, and ionic solution) employed for its extraction from different epidermal appendages (mainly, feathers and wool, but also nails, claws, beak, hair, or horns) [109]. The amino acid composition may vary depending on the source as well as on the animal breed or diet [110], being the cystine content usually high [111,112,113,114]. Transparent materials have been obtained primarily by casting, resulting in water-sensitive films with adequate UV barrier properties and thermal stability up to 200 °C [109,114]. The toughness of these materials can be enhanced through crosslinking [109].

### 2.5. Microalgae from Sewage Plants

Different microalgae species can completely remove nitrogen and phosphorus from wastewater, being a useful tool for sewage plants [115]. These aquatic microorganisms possess a high protein percentage, with species like *Arthrospira platensis* or *Chlorella* sp. containing around 55 of protein in dry weight, and a lysine content similar to that of soy protein (~3.5%) [43]. Moreover, they offer the advantage of not requiring any soil to develop and allowing the use of nonpotable water as a culture medium when grown in wastewater [116]. The use of microalgae in plastic materials production is also interesting as scalable production seems to be more cost-effective because no prior treatment is needed before their processing [117]. Bioplastics materials have been obtained from microalgae biomass, although mainly blended with petroleum plastics or bioplastics [118,119].

## 3. Processing of Bio-Based Materials

The production of biodegradable materials is one of the most promising and studied pathways to handle the extremely high amount of biowastes and byproducts that the agri-food industry produces every year [58,97,120,121,122,123]. In this sense, the processing techniques and parameters selected strongly influence the end-use of the material developed. Commonly, these new materials are processed by traditional techniques used for synthetic plastic. However, a specific redesign is needed since these green materials require a different range of processing parameters due to their different composition and properties [124] which would influence their final characteristics, which definitely should be different from those of common synthetic materials [125,126].

The processability of a protein-based raw material is typically achieved either by its solubilization in an adequate solvent followed by a wet technique (casting or electrospinning) [127,128,129] or by a dry technique (e.g., extrusion and injection molding), which previously requires its blending with a low-molecular-weight component acting as plasticizer [130]. In the latter case, the plasticizer content is important for optimum control of the processing parameters, which are crucial to modulate the final properties of the materials. Furthermore, the amount of plasticizer alters the glass-transition temperature (T_g_), a key processing parameter to consider during its processing [131,132]. The most extensively reported techniques employed in the production of protein based-materials are described in the following subsections: compression molding, injection molding, extrusion, three-dimensional (3D) printing casting, and electrospinning.

### 3.1. Compression Molding

Compression molding has been used since the early twentieth century for manufacturing plastics [133], although its batch process nature has resulted in a major industrial limitation [133]. During compression, the pre-cured or melt polymer is enclosed into a mold cavity and subjected to a large pressure [134]. In many cases, protein is mixed with a plasticizer to obtain blends that are then confined into the mold and compressed [135]. To perform the process correctly, the mold temperature must be slightly higher than the T_g_ of the protein blend. Therefore, temperatures over 60 °C should be generally selected [97,136]. This processing technique does not require high flowability; therefore, the obtained materials can be reinforced satisfactorily with fibers [27,137,138,139,140,141,142,143,144,145]. In literature, numerous studies have used different protein sources with this technique, such as soy [146], gluten [33,147,148,149,150,151,152], cottonseed [153,154], egg white [155], sunflower [156], corn [39,130], or whey [120,157]. As highlighted elsewhere, the properties of the compression-molded materials depend greatly on the processing temperature, which is usually around 100–120 °C [39,130,148]. Pressure, commonly around 10 bar for 2–10 min, is less influential than temperature [120,153,157].

### 3.2. Injection Molding

When intricate complex geometries and/or dimensional precision are required, injection molding is broadly used in polymer manufacturing, provided that the production is at a large scale [124]. This processing technique is commonly carried out through two stages: first, protein flour and the plasticizer (which is essential in this case) are conveniently mixed, and subsequently, blends are introduced into the injector feeding or cylinder, where the sample is heated if required. Then, the injection pressure is applied by means of a plunger, forcing the blend to flow through a nozzle into the mold cavity. After injection, the pressure is reduced and maintained constant for a period required to allow physical and/or chemical crosslinking of protein segments (i.e., the holding stage) [18,158]. The main control parameters are temperature (of the cylinder and mold), pressure, and time (injection and holding) [124]. In these terms, the mold temperature has been pointed out as the most influential parameter in this technique, where pressure exerts a lower impact [159]. Several studies have highlighted that changes in the mold temperature (and/or in the holding time at which the blend is exposed) can modulate the final properties of the product [17,95,97,121,160], modifying the water uptake capacity and rheological and mechanical properties [32,59,97,159]. Different protein sources have been injection-molded, such as soy [18,58,59,62,161,162,163,164,165,166,167,168], sunflower [77], albumen [155,160], porcine plasma [95,96,97], pea [121,159], whey [144], rice [169], or gluten [32,35,170].

### 3.3. Extrusion

Extrusion is widely employed for the manufacturing of plastics with constant section [171,172,173], with the extra benefit of its continuous processing mode. In this technique, temperature is controlled along a cylindrical barrel containing a screw that allows the mixing and the transport of the material from the hopper to the die. During processing, the protein/plasticizer blends combine thermoplastic behavior with heat-induced crosslinking of different nature (e.g., protein aggregation and disulphide bonds). The selection of the temperature profile along the barrel is extremely important [174]. Therefore, a previous thermal characterization of the blend is helpful, using the T_g_, which depends on the glycerol content [34,175], as the temperature threshold that must facilitate the flow inside the extruder and through the die [176]. Commonly, lower temperatures are required for protein-based blends, due to their usually lower T_g_ and to avoid massive crosslinking that would impede the process [171,174]. Furthermore, shear impact, time, and specific mechanical energy have been also pointed out to be key parameters to control the extrusion process [177,178]. At the extruder die, the material is conveniently shaped [178] and successively cooled down [172].

The protein more extensively processed through this method has been wheat gluten [34,36,171,177,178,179,180,181,182,183], followed by soy protein [184,185,186,187,188,189].

#### 3D-Printing

In the most extended 3D technique (fused deposition modeling, FDM) for the automated and additive manufacturing of 3D objects, after layer-by-layer deposition, no excessive equipment investment is required [190], and relatively low energy is demanded per batch [158]. However, its main competitive drawback is referred to the time required for great productions [191]. This strategy has been developed fundamentally for polymeric materials, and it has been used in different fields such as biomedicine [191,192], electronic [193,194], or food [195,196], among others that typically involve small-scale production and high-value-added products. Although this manufacturing strategy has been scarcely exploited using protein-based products, some studies have been developed using pea [197], plasma [198], soy [199], and milk proteins [200,201]. All these studies highlighted the importance that rheology exerts on the 3D printing process. Thus, the main control parameters are those which exert influence over the rheology, such as temperature and shear rate (related to the flow through the nozzle).

### 3.4. Casting

The processing of protein-based biowastes into bioplastics films by casting is the most reported strategy, with applications as coating or packaging extensively described [127,202,203], despite generally possessing lower mechanical properties than those of synthetic materials [128,204]. It is a wet processing technique in which a prior disruption of linkages and disulphide bonds is carried out through a chemical reagent [205]. Then, the protein source is solubilized in a proper solvent, along with the plasticizer and other components such as crosslinking or antimicrobial agents. To produce the protein-based film, the solution is first spread, and then the solvent evaporation or drying is produced [127,206]. This procedure is mainly controlled by the pH and temperature of the solution, as well as by the selected solvent [206]. Based on its high content in cysteine residues, which are key since they promote covalent bonds [207], gluten is the protein most employed to give rise this kind of biodegradable films [207,208,209,210,211,212,213,214,215,216,217,218,219,220,221,222,223,224,225]. Furthermore, several studies were aimed at obtaining films of protein-based materials by casting, such as zein corn [226,227,228,229], soy [61,230,231], milk [232,233,234,235,236], sunflower [79], pea [237,238,239,240], and fish [205,217,241,242,243,244].

### 3.5. Electrospinning

The electrospinning technique produces nanofibrous polymeric materials using a high-voltage electric field [245,246,247]. The polymeric solution is confined in a syringe and flowed out through the needle using a syringe pump. When an electric field acts over the polymeric solution, the so-called Taylor’s cone is formed at the end of the needle [248]. Thus, the produced flow is boosted toward the collector by applying a direct electric current field (commonly from 5 to 25 kV) connected to the collector and needle, which are usually placed away at a distance of 10–20 cm [249,250]. Electrospun materials are formed by ultrathin fibers with diameters in the nanoscale and commonly possess low density and a high porosity, resulting in materials with large specific surface areas [251]. If a certain degree of alignment is required for the nanofibers (e.g., biomedical functions), a rotating collector should be employed during their production [252,253,254]. The main parameters to control the morphological characteristics of the fibers formed are the type of polymer and solvent used, the surface tension, the viscosity of the solution, the flowrate, and the voltage applied [255]. Other parameters that may affect the process are the electrical conductivity, the presence of electrostatic interactions, and the distance between the needle and the collector.

Although it is difficult to carry out the electrospinning of protein solutions, they could be denatured to some extent to induce the process [251,256,257,258]. For aqueous protein solutions, pH is also a key parameter since it may modulate the charges of protein surfaces and protein solubility. Several studies have been focused on the electrospinning of protein solutions using gelatin [246,259,260,261], soy [262], egg albumen [263], silk fibroin [264], or whey protein [265].

## 4. Characterization of Protein-Based Materials

Any material processed for any purpose (e.g., packaging, coating, and agricultural) must reach some specific characteristics to properly provide the functionality required. Thus, the mechanical properties, the thermal behavior, and/or the optical properties of these materials should be controlled to meet the requirements of their final use. Characterization techniques quantify the macroscopic parameters, relating them to the microscopic (even molecular) structure of the materials. The most important characterization techniques are explained in the following subsections.

### 4.1. Mechanical and Rheological Properties

Mechanical properties help to understand and predict the behavior of materials subjected to different kind of stresses. The majority of polymer-based materials show a variety of viscoelastic responses after an applied strain or stress. When tested, materials are typically submitted either to continuous or oscillatory deformation. It can be noted that only some of the most important tests for material characterization are described in this section.

#### 4.1.1. Tensile Strength Tests

During these tests, the material is subjected to axial deformation at a constant rate until breakdown. The results are plotted in stress–strain curves where three different stages can be typically differentiated in polymer-based materials: (i) Initially, the strain suffered by the material is linearly proportional to the stress applied, due to the elastic deformation of the material. From this initial constant slope, the Young’s modulus (E) is defined. (ii) Subsequently, a remarkable decrease in the slope takes place, showing a nonelastic deformation. The maximum value or ultimate stress (σ_max_) is commonly reached at the end of this section. (iii) Finally, the stress decreases due to the fast reduction of the cross-sectional surface, ending the test when the probe collapses at maximum deformation (ε_max_). [266]. The mechanical properties of different materials obtained from several biopolymers, such as rice [267], albumen [268], plasma [95,97], soy [160], pea [121], or whey protein [32,120], have been analyzed through this technique. A wide spectrum of values for the mechanical parameters was obtained, depending not only on the source, the biopolymer/plasticizer ratio, and the presence of additives (e.g., a crosslinker) but also on the processing technique and conditions.

#### 4.1.2. Rheological Tests

Rheology is the science that studies the flow and deformation of matter and applies to a wide range of materials [269]. Moreover, its importance is key since the majority of polymeric materials show complex viscoelastic behavior [270], which is the result of the combination of solid-like elastic properties and fluid-like viscous properties [271]. Rheological tests are normally carried out using oscillatory or continuous deformation.

##### Dynamic Mechanical Analysis (DMA)

In these tests, an oscillatory deformation (with a small-strain amplitude, $\gamma_{0}$ ) is applied to the sample, obtaining a sinusoidal stress response with amplitude $\sigma_{0}$ , below the limit for the linear viscoelastic region (LVR). The viscoelastic behavior of the sample is obtained relating both stimulus and response, giving rise to the linear viscoelastic functions that remain independent of the applied strain [270]. The most important linear viscoelastic functions are the storage modulus (E′ or G′), which is a measure of the elastic response of the material; the loss modulus (E″ or G″), representing the viscous properties; and the loss tangent (tan δ, where δ is the phase angle). This parameter represents the relative predominance of the viscous over the elastic properties (tan δ = E″/E′ or G″/G′). The viscoelastic characterization of the material consists of obtaining the dependence of these parameters on frequency and temperature.

Common dynamic mechanical tests are: (i) stress (or strain) sweep tests, which allow the determination of the LVR through the identification of a critical strain value; (ii) frequency sweep tests, which provides information about the unperturbed microstructure of the sample [33,176]; and (iii) temperature sweep tests, in which the dependence of the material on temperature is analyzed. These measurements can be carried by applying different deformation modes (e.g., compression, tension, bending, and shear). The nature of the sample tested would determine the type of geometry and mode that better fits the analysis (parallel plates [272], rectangular [59], dual cantilever [169,273], or three-point bending, among others).

The results of these tests could give relevant information that can be related to processing parameters of the materials [97,274] or even predict some correlations with other properties, such as printability [198] or biodegradability [275]. This rheological characterization has been largely performed for different protein-based bioplastics, such as soy [58], plasma [95,198], zein [276], or pea [121,159], among others [277,278].

##### Continuous Deformation Tests

Stress relaxation and creep tests could be regarded as the most useful long-term assays using continuous deformation. Stress relaxation tests record the evolution of stress until it reaches a plateau while applying a constant strain $\gamma_{0}$ . On the other hand, creep tests apply a constant stress $\sigma_{0}$ while measuring the progressive deformation of the sample.

Within the LVR, a relaxation modulus (G(t)), defined as the ratio between stress (σ(t)) and strain (γ_0_), and a compliance modulus (J(t)), defined as the ratio between deformation (γ(t)) and stress (σ_0_), are defined for relaxation or creep tests, respectively. Representative relaxation and retardation times may be defined from these tests. Creep tests have been used to identify the crosslinking degree by glutaraldehyde in gelatin-based materials [279]. Furthermore, soy-based [280] and fish-based materials [217] have been rheologically characterized through these kinds of tests.

### 4.2. Thermal Characterization

The knowledge of the thermal events when samples are subjected to changes in temperature is quite relevant to identify a suitable end-use of the material. Glass-transition temperature (T_g_) should be identified to properly set the processing parameters of techniques involving heating/cooling of the sample [183]. T_g_ is the temperature above which the mobility of polymeric chains increases prominently as secondary interactions disappear, resulting in biopolymer conformational changes [174,281]. This temperature depends on the nature of the biopolymer (e.g., amino acid sequence) and the amount of plasticizer used in the formulation [282].

The most extensively used tests to measure the changes induced by temperature in materials are differential scanning calorimetry (DSC), thermogravimetric analysis (TGA), and dynamic mechanical thermal analysis (DMTA).

#### 4.2.1. Differential Scanning Calorimetry (DSC)

The main thermal events observed through DSC in protein-based bioplastics are protein denaturation and glass-transition temperature. They could be clearly identified in a thermogram since denaturation is an irreversible event commonly recognized as a minimum in the curve (exo-up type plot). On the other hand, a reversible glass transition is showed as a change in the slope or an inflection point. Additionally, protein aging prior to processing can be also determined by DSC, being identified as a minimum in the curve, but at a much lower temperature than the denaturation temperature [283]. Thus, two scans are typically performed for separating reversible and irreversible thermal events. Although the irreversible events (denaturation) are only shown in the first scan, the reversible events (glass transition) are always observed. Physical aging, being a reversible event, requires longer times and is normally not observed in the second scan neither. This assay is applied to protein-based powder, blends, or materials [17], and it has been used to characterized microalgae protein [284], soy protein [285], wheat gluten [33,149,286], egg yolk [287], pea protein [159], plasma protein [97], canola protein [74], and whey protein [288], among protein systems used for the bioplastic formation.

#### 4.2.2. Thermogravimetric Analysis (TGA)

TGA measurements can be used to determine the thermal stability of protein/plasticizer blends and bioplastics since it measures the weight reduction of a sample as the temperature increases. Several regions can be observed in protein-based bioplastics. Thus, below 150 °C, the diminution in the weight is caused principally by the loss of volatile components and water [283,289]. At higher temperatures (c.a., 180–350 °C), the weight loss has been attributed to protein degradation [290]. Moreover, these thermograms can indicate the fat content of isolated proteins [125], the presence of volatile components after processing [144], or the response of some active ingredients such as citric acid when used in the production of soy-based porous materials [58]. Moreover, this technique has also been used to determine the thermal stability of protein-based materials after the acylation of the protein [163].

#### 4.2.3. Dynamic Mechanical Thermal Analysis (DMTA)

This technique measures the rheological response of a material as a function of temperature, after application of a small-amplitude oscillatory strain (or stress), as previously commented in Section 4.1.2. This technique relates the evolution of the viscoelastic moduli of biopolymers with temperature to their molecular structure, giving complementary information to that obtained from DSC and TGA analysis. T_g_ can also be determined from DMTA tests, although its value is dependent on the heating rate and the technique employed [183,291].

Several protein-based materials have been tested through DMTA, such as materials obtained from egg albumen [155,268], soy [17,59], microalgae [284], pea [121], wheat gluten [33,149,290], rice [169], bloodmeal [92], plasma [97,292,293], and canola [76], among others.

### 4.3. Morphological Properties

Microscopy is widely applied to bioplastics to analyze their morphology. Scanning electron microscopy (SEM) is the most widely used for the materials and matrices based on a wide variety of proteins, such as soy [17,18,165], pea [121], wheat gluten [33,149,150], rice [169], or plasma [96,292]. Furthermore, the surface of bioplastics films could be also studied by SEM [294,295]. On the other hand, when transmission electron microscopy (TEM) is used some considerations have to be made as this technique requires electron transparency, which may be acquired directly by thin bioplastic film [296] or by cutting thin slides from bioplastic probes [185,297]. Additionally, atomic force microscopy (AFM) is useful to study the topography of the three-dimensional surface of protein-based materials [155,296]. Finally, confocal laser scanning microscopy (CLSM) can exploit the autofluorescence of certain proteins, being quite useful to characterize the morphology of protein-based films [298].

### 4.4. Optical Properties

The optical properties, such as color, transparency, or refractive index, may be sometimes neglected, but they are quite important for several end-uses of these materials. They are highly dependent on the nature of the protein source [17], the composition of the system, the amount of plasticizer [121], and the processing technique or conditions [159,299]. Transparency has been used, for instance, to quantify the presence of some microbial polysaccharides in gluten WPI-based films [300].

## 5. Applications of Protein-Based Bioplastics

The main agri-food industrial biowastes and co-products for bioplastic applications has been described in the previous section. Food biowastes can be used for the production of biofuels [301], but the present review focuses on their application in the field of greener materials, which has been extensively studied but is less exploited commercially, especially the protein fraction. The selection of a suitable biopolymer source is key in the development of any final product with a particular application, which may be chosen based not only on its processing suitability but also on consumer requirements. For instance, animal proteins are commonly rejected in cosmetics, despite being widely accepted in agricultural applications [302]. Moreover, the design and development of bioplastic materials need to bear in mind the accordance between service conditions and the final mechanical and functional properties of the material developed. Although many researchers focus on the mechanical properties of bio-based bioplastics, many applications (i.e., superabsorbent, drug delivery, controlled release, etc.) do not require excellent mechanical properties for their final usage [18,97]. Some critical requirements are demanded for these bio-based materials when used in food applications. For instance, food quality and safety during storage should not be compromised. Moreover, extended shelf-life and a reduced permeability to volatile compounds (i.e., oxygen and moisture) are also pursued [303]. This section summarizes the main applications for the agri-food industry biowastes whose end-use can be linked to the goals of the bioplastic industry. Moreover, this section also addresses the requirements of these bio-based materials for certain applications.

### 5.1. Food Packaging and Coating

Apart from the specific safety and security requirements in food applications, the new generation of packaging materials aims at biodegradability, in order to avoid accumulative pollution, together with advanced extra features [304,305]. The requirements in food packaging and coating depends on the nature of the food contained [306]. For instance, to extend the expiration date of vegetables, respiration and transpiration rates must be reduced during storage (i.e., controlling temperature, relative humidity, light, and gas permeation) [307]. However, these requirements should be adapted for every specific food application. Dairy products are mainly degraded by oxidation and microbial growth, leading to nutrient loss, which causes color changes, as well as the appearance of undesirable flavors [308]. Analogous effects are observed in meat products, where the CO_2_ and O_2_ levels should be kept in a suitable range [309]. Eventually, a wide variety of food products are maintained frozen during its conservation, regardless of the nature of the food matrix. Although low temperatures prevent microbial growth, a suitable package design for food preservation is still required when freezing food products. Light and oxidation promote the degradation of vitamins and pigments, destabilization of proteins, and oxidation of lipids. Thus, package design should avoid those phenomena. Additionally, packages for frozen food products should avoid moisture loss by water sublimation (i.e., low water permeability), which results in undesirable consequences such as weight loss, the appearance of burns, and morphology changes [310].

These undesirable phenomena in different types of food products have been so far commonly overcome by the use of synthetic polymers. However, the food packaging industry is facing a challenge in providing an adequate solution for correct food conservation and environmental sustainability. In this sense, zein has been used for the preservation of tomatoes, avoiding color changes [311]. This protein has been also used for fruits, showing a reduction in weight loss, which is something expected from every coating [312]. Zein films have been also used for extending the shelf-life of dairy products, reducing protein oxidation [313]. Soy protein has been used in the development of coatings which prevent peanut deterioration [314]. Gelatin has been extensively used for the manufacturing of protein films with coating applications for the conservation of fresh products (both meats and vegetables) [315,316,317,318]. Protein biowastes from the dairy processing industry have also been used for the manufacture of films. Thus, sodium caseinate and whey protein concentrate were the most common proteins for a new generation of films for food applications not only in dairy products, such as cheese, but also in other food products, such as meat [319,320,321]. Keratin has been used for the coating of meat derivatives, exhibiting good properties for the formation of films [322]. Eventually, as protein hydrophobicity is not enough to avoid the water permeability required for some applications (e.g., frozen products), improvements should be addressed to reduce the abovementioned side-effects of water loss [304]. In any case, suitable values were obtained for frozen fishes [323,324].

Most functionalities of biodegradable protein-based materials find applications in food active-packaging, including the controlled release and immobilization of substances for specific purposes (i.e., antioxidant release, enzyme activity, gas selective permeability, etc.) [325]. Active packages are able to protect and interact with the food they contain by “deliberately incorporating components that would release or absorb substances into or from the packaged food or the environment surrounding the food” [326]. Thus, these materials can be considered as active packaging since there is an interaction between the material and the food product contained including activities such as controlled release, which improve its preservation [327,328]. Although some alternatives from synthetic polymers have been proposed, bio-based active packaging also offers the advantage of avoiding phthalate leaching typically produced in synthetic polymers [329]. Thus, the evaluation of active-agent release from biopolymers has received outstanding attention from some years ago, with promising applications in the food packaging industry [152]. More specifically, the efficiency of active packaging for antimicrobial activity is based on the match between the releasing rate of the active agent and the decrease in the growth kinetics of the target microorganism [330]. These innovative materials can extend the shelf-life of food products, providing an increase in microbial safety. This increase in food security can be achieved by the incorporation of antimicrobial agents into the bulk of the biopolymer matrix or onto the biopolymer surface [331,332]. Several authors have used co-products from the dairy industry with antimicrobial constituents (i.e., oregano, rosemary, and garlic essential oils) for the manufacturing of edible WPI-based antimicrobial films [332]. This same application was found for other protein-based materials such as zein [333] and soy protein isolate [333] by adding a mixture of lysozyme and nisin. However, other active agents can be also used. To this end, Redl et al. [334] added ascorbic acid to confer antimicrobial properties to gluten-based bioplastics. Gelatin was also mixed with chitosan to confer antimicrobial properties [335]. Another biofunctional property, which is typically required in food packaging, is the antioxidant activity. More fresh products are demanded nowadays by consumers [336]. However, as abovementioned, the lipid oxidation reactions bring undesirable off-odors and off-flavors, as well as changes in texture and color [337]. Moreover, apart from physical changes, lipid oxidation can also generate toxic compounds such as aldehydes [338]. However, the use of synthetic antioxidants such as butylated hydroxytoluene (BHT) and butylated hydroxyanisole (BHA) should be avoided since they have been related to undesired health side-effects [339]. Gelatin films have been developed together with citronella oil to manufacture films with antioxidant properties [340]. The release of carotenoids from seaweeds with proved antioxidant activity in synthetic packaging [341] suggests that the manufacture of algae-based bioplastics might be considered to replace the synthetic polymer if the processing method selected do no alter them. Moreover, it can be also indicated that whey and soy protein isolates have been used in propylene-based materials to confer antioxidant properties [342] to them, including even antifungal ones [343].

### 5.2. Absorbent and Superabsorbent Materials (SAMs)

Some absorbent materials exhibit an exceptional water absorption capacity. They are named superabsorbent materials (SAMs), provided that they can absorb an amount of water greater than 1000% their own weight [344]. The capacity of SAMs to hold a huge amount of water is based on their ability to convert their structure into a hydrogel which is not dissolved when the solvent (i.e., water in most cases) is trapped onto the 3D network [345]. The synthesis of the first fossil-based superabsorbent polymer dates back to the late 1930s, while the first commercial SAM was marketed in 1978 [346]. However, these synthetic polymers show very low biodegradability, and hence, their replacement would lead to a positive reduction in the environmental impact of SAMs [347]. Carbohydrates are key biopolymers in the manufacture of this type of materials [348,349,350], but many researchers have also focused their investigations on protein biowastes and co-products from the agri-food industry to generate biodegradable absorbent and superabsorbent materials [97,123,351,352]. Different processing techniques have been proposed for the manufacture of protein-based absorbent and superabsorbent materials, covering from injection molding [58,59] to casting [353]. The most important applications for these materials are related to personal care and agriculture [354,355]. However, these materials are also applied in the food industry, especially in the control of moisture during food storage and preservation [328]. The interest in SAMs from the food industry is reflected by the publication of several patents aimed to use absorbent materials for food packaging [356,357,358]. In this sense, zein has been used for the synthesis of superabsorbent hydrogels, showing applications as metal ion chelators, which in turn can avoid food oxidation [359]. Moreover, co-products from the cattle industry have also been used as raw materials for the generation of superabsorbent materials for food applications, such as whey and casein [360,361,362].

Due to a lower added value, the agricultural applications reported in the literature are lower. Proteins with low-value applications such as keratin and potato protein concentrates were proposed for the development of superabsorbent materials to be used in agriculture [113,123]. More details about this end-use are shown in the following Section 5.4.

Moreover, the final end-use application is not indicated in many cases, as authors developed SAMs with any final end-use, which includes food packaging, agriculture, personal care, and even scaffolds for cell growth, among others. In this sense, soy protein isolate has been widely used for the manufacture of these materials [58,59,353]. Apart from soy protein, other proteins co-products (i.e., canola and rapeseed protein concentrates) were also used for absorbent materials [76,113,363]. Additionally, plasma protein, a hydrophilic co-product from the meat processing industry, has proven to be useful in the fabrication of absorbent plastic films [91,364,365,366] and superabsorbent materials obtained by injection molding [96] and even 3D printing [198]. Also from the meat industry, gelatin showed suitable application as SAMs [367]. Eventually, the underutilized bio-resource marine algal seaweeds can be also converted into added-value superabsorbent materials. Thus, apart from the formation of hydrogels with κ-carrageenan and alginate [368,369], the protein fraction from micro- and macro-algae is suitable for the generation of absorbent materials [119,284].

Furthermore, soy proteins have also been chemically modified, improving the superabsorbent properties of the materials produced by the acylation with ethylenediaminetetraacetic dianhydride (EDTAD) or succinic anhydride [59,62,363]. Other authors have also used EDTAD to functionalize other cereal proteins, like wheat gluten, improving the initial water absorption properties of this co-product [370].

### 5.3. Agriculture

Polymers have found some interesting applications in agriculture, such as controlled release of specific micronutrients or pesticides, as porous and water-holding matrices that avoid the critical soil drought. Moreover, some of these applications have been linked to the superabsorbent ability of the polymers employed. In the 1980s, several companies manufactured and commercialized composites materials based on plant biopolymers and polyethylene aiming at simultaneously providing biodegradability and fulfilling the requirements of the consumers. However, this strategy resulted in the release of polymer chains and microplastics to the medium, which caused environmental damage due to the toxic polyethylene residues [371]. In this way, microplastics disposed on land end up in rivers that flow into seas, eventually contaminating aquatic environments [372]. Therefore, the reduction of sea microplastics must go through the reduction of inputs onto the inland [373]. Microplastics are particles lower than 5 mm built from larger particles pieces. These particles cause damage to marine species, which eventually may affect the full food chain [374]. The removal of these small bodies is a real challenge; therefore, the best option is to avoid its generation [373,375]. Therefore, if the aim is to increase polymer biodegradability for agricultural applications, the path can go through the use of biopolymers whose degradation does not release undesirable macro- and micro-plastics [371]. To this end, superabsorbent biodegradable polymers have been proposed for their use in water-saving applications as well as for the controlled release of essential nutrients for plants [376,377]. These superabsorbent materials increase the overall porosity of clay-based soils, being recommended for their use in dry agricultural areas to reduce the drought stress during plant growth [378]. Moreover, plant nutrients may also be entrapped within superabsorbent matrices for their controlled release, hindering the water losses due to evaporation, and reducing the irrigation [378]. Although there are polysaccharide-based materials for these applications [379,380,381], Capezza et al. [370] recently reported the use of gluten co-product as raw material for the manufacture of protein-based superabsorbent materials with agricultural applications. These authors used a crosslinking agent (i.e., genipin) together with EDTAD to improve probe swelling, which in turn increased the water absorption capacity of these materials. Moreover, SAMs with agricultural applications have been generated from other proteins, such as soy [59], canola [363], zein [382], blood plasma [95], keratin and gelatin [383], milk proteins [384,385], and seaweed [347,386]. Moreover, bioplastics can also be used for the controlled release of both micronutrients and pesticides. This is the case of gluten-based bioplastics (releasing pesticides) [387], soy-based bioplastics (releasing zinc and mineral nutrients) [388,389], or even zein to prevent salt-leaching [390]. These results evidenced the potential of co- and by-products from the agri-food industry to produce materials that can be reused in agricultural applications with greater environmental performance. Accordingly, the life cycle of some crops may be enhanced since they can be used to obtain co- and by-products that in turn may lead to bioplastics that could be reused again in the first stages of their own life cycle (e.g., as water suppliers or for nutrient delivery to enhance crop growth).

### 5.4. Scaffolds

Scaffolds are temporary supporting structures which are used to generate the final structure. In material science, scaffolds are typically used for tissue engineering. Thus, although the properties typically found in scaffolds materials (e.g., high porosity and water absorption) can confer them interesting features, they are only named scaffolds when serving as supporting structure to facilitate cell growth for a certain period of time in tissue engineering [391]. Consequently, scaffolds need to play four fundamental functions: (i) form a complex structure which allows cells to rebuild the original 3D structure; (ii) be temporal support of functional demands (e.g., mechanical support); (iii) enhance tissue regeneration (i.e., the release of bioactive compounds which allow cell fixation and growth, favoring their transport); and (iv) be able to be attached to the surrounding tissue [392,393]. The exposed surface is key for these materials since the larger the surface available, the more cell interactions take place. Thus, the pore size and morphology can significantly influence the performance of these materials, where optimum values should be found for different surrounding microenvironments (where cell dimension also must be considered) [261,392].

The research and further development of protein-based scaffolds have been focused on films, plastics, foams, gels, and even composites materials [394]. Although these materials are developed for tissue engineering, other biomedical applications have been found, such as drug delivery systems and biosensors [395].

Collagen/gelatin has been widely selected as raw material for the manufacture of scaffolds, since it not only performs a supporting function but it is also involved in a wide range of tissue functions [396]. Therefore, collagen has shown excellent properties in scaffolds, being manufactured in different ways such as phase separation [397,398] or electrospinning [399]. Moreover, other proteins have also been used for the development of scaffolds. Fibrous membranes produced by electrospinning were obtained using PLLA/keratin as raw material [400]. In this case, the presence of keratin facilitated the interactions between osteoblasts and the membrane, favoring cell growth. Nanocomposites from keratin/hydroxyapatite have been generated following a co-precipitation method, showing good biocompatibility tested by in vitro tests [401]. Keratin has also been used in combination with natural polymers (i.e., gelatin and chitosan) for tissue engineering applications [402]. In this context, plant proteins (e.g., zein, soy protein, and wheat gluten) can also be used as raw material for scaffolds. They provide suitable mechanical properties, while at the same time being biocompatible. Moreover, their typically low solubility confers them enough stability in aqueous media to be considered appropriate for tissue engineering applications [403]. Eventually, some authors have also tested the stability of seaweeds for the manufacture of scaffolds, being used in most cases in combination with other polymers such as PLA and cellulose [404,405]. All these results evidence that protein biowastes and co-products from the agri-food industry can even be employed on specific applications such as scaffolds for cell growth.

### 5.5. Other Applications

Although the most important applications for bioplastics from co- and by-products from the agri-food industry have been mentioned above, other applications can be proposed. Nanoparticles from collagen-serum albumin composite have been used for drug delivery [406]. In the biomedical field, keratin has been used as nanosuspension to analyze cell proliferation in tissue engineering applications (as an alternative to fibronectin and collagen, typically used for this purpose) [407]. Although some authors have investigated the use of protein-based bioplastics in the textile industry, proteins typically lead to bioplastics exhibiting a fairly low elongation and are therefore brittle, which do not convert them into suitable raw material for textile applications [408]. This is not the case of the automotive industry, where Mohanty et al. [162] proposed the use of soy-based composite materials (reinforced with fibers). This same approach was found by Guilbert et al. [206] when soy-based bioplastics were hardened with formaldehyde. Saenghirunwattana et al. [145] proposed the use of a zein protein concentrate for the manufacture of composite materials with applications in construction. Even electrical properties (dielectric constant) have been analyzed and modulated in soy-based bioplastics for electrical applications [409].

Main protein sources from biowastes together with the processing technique used and application pursued are summarized in Table 1.

## 6. Future Trends

Findings gathered in the present review put into focus the wide versatility of bioplastics manufactured from agri-food industrial biowastes or co-products, although the limits for their applicability are still far from being fully explored by the scientific community. In the relatively near future, conventional plastics will disappear in single-use applications, following the European strategy for plastics in a circular economy, which aims to transform the way plastic products are designed, used, produced, and recycled in the European Union. Most current applications are focused on the use of lignocellulose, starch, or fats from food biowaste, and the protein fraction is mostly relegated to low-value applications (e.g., animal food). However, as highlighted in the several applications described above, there is a solid scientific ground to industrially exploit those protein-rich biowastes and co-products. Techniques like electrospinning or 3D-printing have yet to further develop their potential to do so, and proteins which are noncompetitive with the agri-food industry, such as rapeseed or keratin, may find a privileged position. However, the excess of co-products that are only minimally used by the agri-food industry despite being edible, such as blood from the meat industry, should be better employed in applications like those herein presented, in agreement with a circular economy. When bioplastics generated from biowastes and co-products such as those herein indicated are competitive, the laws of supply and demand will help to modulate their use.

## Figures and Tables

**Table 1:** Main protein sources from biowastes used in the development of plastic materials commented in the present review.

Source	Protein	Processing Technique	Plasticizer/Solvent or Carrier	Application	References
	Wheat gluten	Acylation	glycerol	Superabsorbent materials	[34]
		Compression moulding	glycerol	Horticulture (release of pesticides)	[276]
		Compression moulding	glycerol	Packaging	[29,154]
		Compression moulding	Water/glycerol	Edible films	[153]
		Compression moulding	glycerol	Biodegradable films	[275]
		Compression moulding/Injection moulding	glycerol	Disposable articles	[171]
Starch		Casting	glycerol	Food packaging films	[162]
		Casting	glycerol	Disposable articles	[210]
		Casting	glycerol/ethanol	Edible films	[223]
		Extrusion/Injection moulding	glycerol	Superabsorbent materials	[32]
		Extrusion	glycerol	Disposable articles	[34]
		Extrusion	glycerol	Superabsorbent materials	[36]
	Potato	Acylation	glycerol	Superabsorbent materials	[120]
		Casting	Ethylene glycol, propylene glycol, glycerol, sorbitol and polyethylene glicol	Food packaging films	[46]
Bioetanol	Zein	Compression and casting	glycerol (compression) and glycerol/ethanol (casting)	Antimicrobial packaging films	[333]
		Casting	glycerol/ethanol	Packaging of tomatoes, reduction of color loss	[311]
		Casting	glycerol/ethanol	Apples and pears, reduction of water loss	[312]
		Casting	glycerol/ethanol	Reduction of oxidation in dairy products	[313]
		Casting	Ethanol/Polyols (sorbitol, glycerol and mannitol)	Food packaging films	[227]
		Extrusion	water/ethanol	Food packaging films	[51,52]
Oil	Soy	Casting	water	Edible films	[230]
		Casting	water and glycerol	Edible films	[227]
		Extrusion	glycerol	Disposable articles	[185,189]
		acylation-Injection moulding	glycerol	Superabsorbent materials	[60,62,163]
		Injection moulding	glycerol	Superabsorbent materials	[18,58,59]
		Injection moulding	glycerol	Horticulture (Zn incorporated)	[63]
		3D printing	water/gelatine and sodium alginate	Food matrix	[199]
	Canola/Rapeseed	Casting	glycerol	Edible films	[72,73]
		compression moulding	polyvinyl alcohol and glycerol	Disposable articles	[75]
		injection moulding	glycerol	Packaging	[76]
		casting	glycerol, 1,3-propanediol, D-sorbitol, triethylene glycol, tetraethylene glycol	Films	[79]
	Sunflower	compression moulding	glycerol	Edible films or packaging	[156]
		extrusion/injection moulding	water	planting containers	[77]
		extrusion	water and glycerol	Edible films	[80]
Animal farming	Blood	Extrusion	Water	-	[92]
		Injection-moulding	glycerol	-	[93]
	Plasma	Casting	glycerol	Food wrap or coating	[91,365,366]
		Casting	glycerol	Food packaging	[364]
		Injection-moulding	glycerol	Superabsorbent materials	[95,96,97,292]
		3D printing	glycerol	-	[198]
	Keratine	Casting	glycerol, water, SDS	Food packaging, coating, medicine	[109]
		Casting	glycerol, polyethilene	-	[112]
	Gelatine	Casting	Water	Packaging and coating	[279]
		Electrospining	acetic acid and dimethylsulfoxide	Regenerative medicine	[259]
		Electrospining	2,2,2-trifluorothanol	Biomaterials	[288]
		Electrospining	Trifluoroacetic acid	Biomaterials	[260]
		Electrospining	Acetic acid	Tissue engineering	[261]
	Milk protein	Casting	Clycerol, Propylene glycol, sorbitor, sucrose and polyethylene glycol	Coating, food packaging	[233]
		3D printing	Water and sodium caseinate	Costumized food design	[200,201]
	Casein	Casting	glycerol	Packaging	[82]
		Hydrogel by solubilization	Transglutaminase	Contolled release	[361,362]
	Whey	Casting	-	Food coating	[319]
		Casting	sorbitol	Active packaging	[321]
		Casting	glycerol	Food packaging	[232,233,235,332]
		Casting	water	Coating	[234]
		Casting	glycerol	Coating, edible films	[376,380,386]
		Freezing	glycerol and sorbitol	Coating	[324]
		Compression	Water	Food packaging	[145]
		Hydrogel by solubilization	glycerol	Coating, food packaging	[297]
		Electrospining	acetic acid	Coating	[265]
	Fish	Casting	glycerol	Active packaging	[340]
		Casting	glycerol	Edible packaging	[209]
		Casting	glycerol	Food packaging	[241,244]
		Compression moulding	glycerol	Active packaging	[141]
Sewage	Microalgae	Compression moulding	glycerol	Disposable articles	[116]
		Injection moulding	glycerol	Packaging	[118,284]

## Data Availability

All the results showed in the manuscript could be requested to the corresponding author who would provide them.

## References

[1] Rai P., Mehrotra S., Priya S., Gnansounou E., Sharma S.K. (2021). Recent advances in the sustainable design and applications of biodegradable polymers. Bioresour. Technol..

[2] Chamas A., Moon H., Zheng J., Qiu Y., Tabassum T., Jang J.H., Abu-Omar M., Scott S.L., Suh S. (2020). Degradation rates of plastics in the environment. ACS Sustain. Chem. Eng..

[3] Maraveas C. (2020). Production of sustainable and biodegradable polymers from agricultural waste. Polymers.

[4] Bhatia S.K., Otari S.V., Jeon J.-M., Gurav R., Choi Y.-K., Bhatia R.K., Pugazhendhi A., Kumar V., Rajesh Banu J., Yoon J.-J. (2021). Biowaste-to-bioplastic (polyhydroxyalkanoates): Conversion technologies, strategies, challenges, and perspective. Bioresour. Technol..

[5] European Bioplastics (2019). Bioplastics, Facts and Figures.

[6] Acquavia M.A., Pascale R., Martelli G., Bondoni M., Bianco G. (2021). Natural polymeric materials: A solution to plastic pollution from the agro-food sector. Polymers.

[7] Thiruchelvi R., Das A., Sikdar E. (2020). Bioplastics as better alternative to petro plastic. Mater. Today Proc..

[8] Gatto F., Re I. (2021). Circular bioeconomy business models to overcome the valley of death. A systematic statistical analysis of studies and projects in emerging bio-based technologies and trends linked to the SME instrument support. Sustainability.

[9] Mishra B., Varjani S., Parida M., Iragavarapu G.P., Awasthi M.K., Awasthi S.K., Zhang Z., Singh A., Srivastava S., Rathore D., Pant D. (2021). Film based packaging for food safety and preservation: Issues and perspectives. Environmental Microbiology and Biotechnology.

[10] Makhijani K., Kumar R., Sharma S.K. (2015). Biodegradability of blended polymers: A comparison of various properties. Crit. Rev. Environ. Sci. Technol..

[11] Reddy M.M., Vivekanandhan S., Misra M., Bhatia S.K., Mohanty A. (2013). Biobased plastics and bionanocomposites: Current status and future opportunities. Prog. Polym. Sci..

[12] FAO (2013). Food Wastage Footprint Impacts on Natural Resources.

[13] Ravindran R., Jaiswal A.K. (2016). Exploitation of food industry waste for high-value products. Trends Biotechnol..

[14] Chan J.X., Wong J.F., Hassan A., Zakaria Z., Saba N., Jawaid M., Thariq M. (2021). 8–Bioplastics from agricultural waste. Biopolymers and Biocomposites from Agro-Waste for Packaging Applications.

[15] Council N.R. (1994). Polymer Science and Engineering: The Shifting Research Frontiers.

[16] Barbi S., Macavei L.I., Caligiani A., Maistrello L., Montorsi M. (2021). From food processing leftovers to bioplastic: A design of experiments approach in a circular economy perspective. Waste Biomass Valorization.

[17] Félix M., Martín-Alfonso J.E., Romero A., Guerrero A., Felix M., Martin-Alfonso J.E., Romero A., Guerrero A. (2014). Development of albumen/soy biobased plastic materials processed by injection molding. J. Food Eng..

[18] Fernández-Espada L., Bengoechea C., Cordobés F., Guerrero A. (2016). Thermomechanical properties and water uptake capacity of soy protein-based bioplastics processed by injection molding. J. Appl. Polym. Sci..

[19] Tsang Y.F., Kumar V., Samadar P., Yang Y., Lee J., Ok Y.S., Song H., Kim K.-H., Kwon E.E., Jeon Y.J. (2019). Production of bioplastic through food waste valorization. Environ. Int..

[20] Reichert C.L., Bugnicourt E., Coltelli M.-B., Cinelli P., Lazzeri A., Canesi I., Braca F., Martínez B.M., Alonso R., Agostinis L. (2020). Bio-based packaging: Materials, modifications, industrial applications and sustainability. Polymers.

[21] Silviana S., Rahayu P. (2019). Central composite design for optimization of starch-based bioplastic with bamboo microfibrillated cellulose as reinforcement assisted by potassium chloride. J. Phys. Conf. Ser..

[22] Chalermthai B., Ashraf M.T., Bastidas-Oyanedel J.-R., Olsen B.D., Schmidt J.E., Taher H. (2020). Techno-economic assessment of whey protein-based plastic production from a co-polymerization process. Polymers.

[23] Mostafa N.A., Farag A.A., Abo-dief H.M., Tayeb A.M. (2014). Production of biodegradable plastic from agricultural wastes. Arab. J. Chem..

[24] Hunt B.J., James M.I. (2012). Polymer Characterisation.

[25] Stenmarck A., Jensen C., Quested T., Moates G. (2016). Estimates of European Food Waste Levels.

[26] del Mar Contreras M., Lama-Muñoz A., Manuel Gutiérrez-Pérez J., Espínola F., Moya M., Castro E. (2019). Protein extraction from agri-food residues for integration in biorefinery: Potential techniques and current status. Bioresour. Technol..

[27] Ye P., Reitz L., Horan C., Parnas R. (2006). Manufacture and biodegradation of wheat gluten/basalt composite material. J. Polym. Environ..

[28] Mason W.R., BeMiller J., Whistler R.B.T.-S. (2009). Chapter 20–Starch use in foods. Food Science and Technology.

[29] Samarasinghe S., Easteal A.J., Edmonds N.R. (2008). Biodegradable plastic composites from corn gluten meal. Polym. Int..

[30] Rombouts I., Lamberts L., Celus I., Lagrain B., Brijs K., Delcour J.A. (2009). Wheat gluten amino acid composition analysis by high-performance anion-exchange chromatography with integrated pulsed amperometric detection. J. Chromatogr. A.

[31] Loy D.D., Lundy E.L., Serna-Saldivar S.O.B.T.-C. (2019). Chapter 23–Nutritional properties and feeding value of corn and its coproducts. Corn.

[32] Álvarez-Castillo E., Ramos M., Bengoechea C., Martínez I., Romero A. (2020). Effect of blend mixing and formulation on thermophysical properties of gluten-based plastics. J. Cereal Sci..

[33] Jerez A., Partal P., Martínez I., Gallegos C., Guerrero A. (2005). Rheology and processing of gluten based bioplastics. Biochem. Eng. J..

[34] Chantapet P., Kunanopparat T., Menut P., Siriwattanayotin S. (2013). Extrusion processing of wheat gluten bioplastic: Effect of the addition of Kraft Lignin. J. Polym. Environ..

[35] John J., Tang J., Bhattacharya M. (1998). Processing of biodegradable blends of wheat gluten and modified polycaprolactone. Polymer.

[36] Jiménez-Rosado M., Zarate-Ramírez L.S., Romero A., Bengoechea C., Partal P., Guerrero A. (2019). Bioplastics based on wheat gluten processed by extrusion. J. Clean. Prod..

[37] Day L., Phillips G.O., Williams P.A.B.T.-H. (2011). 10–Wheat gluten: Production, properties and application. Handbook of Food Proteins.

[38] Capezza A.J., Lundman M., Olsson R.T., Newson W.R., Hedenqvist M.S., Johansson E. (2020). Carboxylated wheat gluten proteins: A green solution for production of sustainable superabsorbent materials. Biomacromolecules.

[39] di Gioia L., Cuq B., Guilbert S. (2000). Mechanical and water barrier properties of corn-protein-based biodegradable plastics. J. Mater. Res..

[40] Kot A.M., Pobiega K., Piwowarek K., Kieliszek M., Błażejak S., Gniewosz M., Lipińska E. (2020). Biotechnological methods of management and utilization of potato industry waste–A review. Potato Res..

[41] Priedniece V., Spalvins K., Ivanovs K., Pubule J., Blumberga D. (2017). Bioproducts from potatoes. A review. Environ. Clim. Technol..

[42] Refstie S., Tiekstra H.A.J. (2003). Potato protein concentrate with low content of solanidine glycoalkaloids in diets for Atlantic salmon (Salmo salar). Aquaculture.

[43] Gorissen S.H.M., Crombag J.J.R., Senden J.M.G., Waterval W.A.H., Bierau J., Verdijk L.B., van Loon L.J.C. (2018). Protein content and amino acid composition of commercially available plant-based protein isolates. Amino Acids.

[44] Newson W.R., Rasheed F., Kuktaite R., Hedenqvist M.S., Gallstedt M., Plivelic T.S., Johansson E. (2015). Commercial potato protein concentrate as a novel source for thermoformed bio-based plastic films with unusual polymerisation and tensile properties. RSC Adv..

[45] Omrani-Fard H., Abbaspour-Fard M.H., Khojastehpour M., Dashti A. (2020). Gelatin/Whey protein–Potato flour bioplastics: Fabrication and evaluation. J. Polym. Environ..

[46] Schäfer D., Reinelt M., Stäbler A., Schmid M. (2018). Mechanical and barrier properties of potato protein isolate-based films. Coatings.

[47] Shukla R., Cheryan M. (2001). Zein: The industrial protein from corn. Ind. Crops Prod..

[48] Elzoghby A.O., Samy W.M., Elgindy N.A. (2012). Protein-based nanocarriers as promising drug and gene delivery systems. J. Control. Release.

[49] Elzoghby A.O., Elgohary M.M., Kamel N.M., Donev R.B.T.-A. (2015). Chapter 6–Implications of protein- and peptide-based nanoparticles as potential vehicles for anticancer drugs. Protein and Peptide Nanoparticles for Drug Delivery.

[50] Tihminlioglu F., Atik İ.D., Özen B. (2010). Water vapor and oxygen-barrier performance of corn–zein coated polypropylene films. J. Food Eng..

[51] Herald T.J., Obuz E., Twombly W.W., Rausch K.D. (2002). Tensile properties of extruded corn protein low-density polyethylene films. Cereal Chem..

[52] Ha T.T., Padua G.W. (2001). Effect of extrusion processing on properties of zein-fatty acids sheets. Trans. Am. Soc. Agric. Eng..

[53] Santosa F.X.B., Padua G.W. (1999). Tensile properties and water absorption of zein sheets plasticized with oleic and linoleic acids. J. Agric. Food Chem..

[54] Lim S., Jane J. (1994). Storage stability of injection-molded starch-zein plastics under dry and humid conditions. J. Environ. Polym. Degrad..

[55] Food and Agriculture Organization of the United Nations (2018). Global Crop Production Analysis–FAOSTAT.

[56] Yamada M., Morimitsu S., Hosono E., Yamada T. (2020). Preparation of bioplastic using soy protein. Int. J. Biol. Macromol..

[57] Mirpoor S.F., Giosafatto C.V.L., Porta R. (2021). Biorefining of seed oil cakes as industrial co-streams for production of innovative bioplastics. A review. Trends Food Sci. Technol..

[58] Fernández-Espada L., Bengoechea C., Sandía J.A.A., Cordobés F., Guerrero A. (2019). Development of novel soy-protein-based superabsorbent matrixes through the addition of salts. J. Appl. Polym. Sci..

[59] Álvarez-Castillo E., Del Toro A., Aguilar J.M., Guerrero A., Bengoechea C. (2018). Optimization of a thermal process for the production of superabsorbent materials based on a soy protein isolate. Ind. Crops Prod..

[60] Hwang D.-C.C., Damodaran S. (1996). Chemical modification strategies for synthesis of protein-based hydrogel. J. Agric. Food Chem..

[61] Guerrero P., Retegi A., Gabilondo N., de la Caba K. (2010). Mechanical and thermal properties of soy protein films processed by casting and compression. J. Food Eng..

[62] Cuadri A.A., Romero A., Bengoechea C., Guerrero A. (2017). Natural superabsorbent plastic materials based on a functionalized soy protein. Polym. Test..

[63] Jiménez-Rosado M., Perez-Puyana V., Cordobés F., Romero A., Guerrero A. (2019). Development of superabsorbent soy protein-based bioplastic matrices with incorporated zinc for horticulture. J. Sci. Food Agric..

[64] Wanasundara J.P.D. (2011). Proteins of Brassicaceae oilseeds and their potential as a plant protein source. Crit. Rev. Food Sci. Nutr..

[65] Pohl F., Goua M., Bermano G., Russell W.R., Scobbie L., Maciel P., Lin P.K.T. (2018). Revalorisation of rapeseed pomace extracts: An in vitro study into its anti-oxidant and DNA protective properties. Food Chem..

[66] Eskin N.A.M., Przybylski R., Caballero B.B.T.-E. (2003). Rape seed oil/canola. Encyclopedia of Food Sciences and Nutrition.

[67] Sjödahl S., Rödin J., Rask L. (1991). Characterization of the 12S globulin complex of Brassica napus. Eur. J. Biochem..

[68] Monsalve R.I., Villalba M., López-Otín C., Rodríguez R. (1991). Structural analysis of the small chain of the 2S albumin, napin nIII, from rapeseed. Chemical and spectroscopic evidence of an intramolecular bond formation. Biochim. Biophys. Acta Protein Struct. Mol. Enzymol..

[69] Wanasundara J.P.D., McIntosh T.C., Perera S.P., Withana-Gamage T.S., Mitra P. (2016). Canola/rapeseed protein-functionality and nutrition. OCL.

[70] Troise A.D., Wilkin J.D., Fiore A. (2018). Impact of rapeseed press-cake on Maillard reaction in a cookie model system. Food Chem..

[71] Pustjens A.M., de Vries S., Bakuwel M., Gruppen H., Gerrits W.J.J., Kabel M.A. (2014). Unfermented recalcitrant polysaccharide structures from rapeseed (Brassica napus) meal in pigs. Ind. Crops Prod..

[72] Chang C., Nickerson M.T. (2015). Effect of protein and glycerol concentration on the mechanical, optical, and water vapor barrier properties of canola protein isolate-based edible films. Food Sci. Technol. Int..

[73] Shi W., Dumont M.-J. (2014). Processing and physical properties of canola protein isolate-based films. Ind. Crops Prod..

[74] Zhang Y., Liu Q., Rempel C. (2018). Processing and characteristics of canola protein-based biodegradable packaging: A review. Crit. Rev. Food Sci. Nutr..

[75] Patel A., Panchal T., Rudakiya D., Gupte A., Patel J. (2016). Fabrication of bio-plastics from protein isolates and its biodegradation studies. Int. J. Chem. Sci. Technol..

[76] Delgado M., Felix M., Bengoechea C. (2018). Development of bioplastic materials: From rapeseed oil industry by products to added-value biodegradable biocomposite materials. Ind. Crops Prod..

[77] Rouilly A., Orliac O., Silvestre F., Rigal L. (2006). New natural injection-moldable composite material from sunflower oil cake. Bioresour. Technol..

[78] Rouilly A., Orliac O., Silvestre F., Rigal L. (2001). DSC study on the thermal properties of sunflower proteins according to their water content. Polymer.

[79] Ayhllon-Meixueiro F., Vaca-Garcia C., Silvestre F. (2000). Biodegradable films from isolate of sunflower (Helianthus annuus) proteins. J. Agric. Food Chem..

[80] Rouilly A., Mériaux A., Geneau-Sbartaï C., Silvestre F., Rigal L. (2006). Film extrusion of sunflower protein isolate. Polym. Eng. Sci..

[81] Ryder K., Ali M.A., Billakanti J., Carne A. (2020). Evaluation of dairy co-product containing composite solutions for the formation of bioplastic films. J. Polym. Environ..

[82] Chambi H., Grosso C. (2006). Edible films produced with gelatin and casein cross-linked with transglutaminase. Food Res. Int..

[83] Cuq B., Gontard N., Guilbert S. (1998). Proteins as agricultural polymers for packaging production. Cereal Chem..

[84] Peters T., Putnam F.W. (1975). 3–Serum Albumin. The Plasma Proteins.

[85] Gatnau R., Polo J., Robert E., Brufau J. (2001). Plasma protein antimicrobialsubstitution at negligible risk. Feed Manufacturing in the Mediterranean Region. Improving Safety: From Feed to Food.

[86] Sanders B. Global Pig Slaughter Statistics and Charts. https://faunalytics.org/global-pig-slaughter-statistics-and-charts/.

[87] Howell N.K., Lawrie R.A. (1983). Functional aspects of blood plasma proteins. Int. J. Food Sci. Technol..

[88] Dàvila E., Parés D., Cuvelier G., Relkin P. (2007). Heat-induced gelation of porcine blood plasma proteins as affected by pH. Meat Sci..

[89] Márquez E., Bracho M., Archile A., Rangel L., Benítez B. (2005). Proteins, isoleucine, lysine and methionine content of bovine, porcine and poultry blood and their fractions. Food Chem..

[90] Sorapukdee S., Narunatsopanon S. (2017). Comparative study on compositions and functional properties of porcine, chicken and duck blood. Korean J. Food Sci. Anim. Resour..

[91] Nuthong P., Benjakul S., Prodpran T. (2009). Characterization of porcine plasma protein-based films as affected by pretreatment and cross-linking agents. Int. J. Biol. Macromol..

[92] Verbeek C.J.R., van den Berg L.E. (2011). Development of proteinous bioplastics using bloodmeal. J. Polym. Environ..

[93] Adamy M., Verbeek C.J.R. (2013). Injection-molding performance and mechanical properties of blood meal-based thermoplastics. Adv. Polym. Technol..

[94] Reinhard V.C.J., Aaron L., Christopher L.M., Maree H.T. (2017). Processability and mechanical properties of bioplastics produced from decoloured bloodmeal. Adv. Polym. Technol..

[95] Álvarez-Castillo E., Bengoechea C., Guerrero A. (2020). Composites from by-products of the food industry for the development of superabsorbent biomaterials. Food Bioprod. Process..

[96] Álvarez-Castillo E., Bengoechea C., Guerrero A. (2020). Effect of pH on the properties of porcine plasma-based superabsorbent materials. Polym. Test..

[97] Álvarez-Castillo E., Bengoechea C., Rodríguez N., Guerrero A. (2019). Development of green superabsorbent materials from a by-product of the meat industry. J. Clean. Prod..

[98] Hicks T.M., Verbeek C.J.R., Dhillon G. (2016). Chapter 1–Protein-rich by-products: Production statistics, legislative restrictions, and management options. Protein Byproducts. Transformation from Environmental Burden into Value-Added Products.

[99] Ghaly A.E., Ramakrishnan V.V., Brooks M.S., Budge S.M., Dave D. (2013). Fish processing wastes as a potential source of proteins, amino acids and oils: A critical review. J. Microb. Biochem. Technol..

[100] Svenson J., Walallavita A.S., Verbeek C.J.R. (2013). Evaluation of fishmeal as starting material for producing biodegradable protein-based thermoplastic polymers. Waste Biomass Valorization.

[101] Mullen A.M., Álvarez C., Pojić M., Hadnadev T.D., Papageorgiou M., Galanakis C.M. (2015). Chapter 2–Classification and target compounds. Food Waste Recovery.

[102] Noorzai S., Verbeek C.J.R., Lay M.C., Swan J. (2019). Collagen extraction from various waste bovine hide sources. Waste Biomass Valorization.

[103] Albaugh V.L., Mukherjee K., Barbul A. (2017). Proline precursors and collagen synthesis: Biochemical challenges of nutrient supplementation and wound healing. J. Nutr..

[104] Lestari W., Octavianti F., Jaswir I., Hendri R. (2019). Plant-based substitutes for gelatin. Contemporary Management and Science Issues in the Halal Industry.

[105] Chentir I., Kchaou H., Hamdi M., Jridi M., Li S., Doumandji A., Nasri M. (2019). Biofunctional gelatin-based films incorporated with food grade phycocyanin extracted from the Saharian cyanobacterium *Arthrospira* sp.. Food Hydrocoll..

[106] Lacroix M., Cooksey K. (2014). Edible films and coatings from animal-origin proteins. Innov. Food Packag..

[107] Murrieta-Martínez C.L., Soto-Valdez H., Pacheco-Aguilar R., Torres-Arreola W., Rodríguez-Felix F., Márquez Ríos E. (2018). Edible protein films: Sources and behavior. Packag. Technol. Sci..

[108] Al-Tayyar N.A., Youssef A.M., Al-hindi R. (2020). Antimicrobial food packaging based on sustainable Bio-based materials for reducing foodborne Pathogens: A review. Food Chem..

[109] Fernández-d’Arlas B. (2019). Tough and functional cross-linked bioplastics from sheep wool keratin. Sci. Rep..

[110] Rajabinejad H., Zoccola M., Patrucco A., Montarsolo A., Rovero G., Tonin C. (2017). Physicochemical properties of keratin extracted from wool by various methods. Text. Res. J..

[111] Tesfaye T., Sithole B., Ramjugernath D. (2017). Valorisation of chicken feathers: A review on recycling and recovery route–Current status and future prospects. Clean Technol. Environ. Policy.

[112] Barone J.R., Schmidt W.F. (2006). Compositions and Films Comprosed of Avian Feather Keratin. U.S. Patent.

[113] Shi W., Dumont M.J. (2014). Review: Bio-based films from zein, keratin, pea, and rapeseed protein feedstocks. J. Mater. Sci..

[114] Ramakrishnan N., Sharma S., Gupta A., Alashwal B.Y. (2018). Keratin based bioplastic film from chicken feathers and its characterization. Int. J. Biol. Macromol..

[115] Martínez M.E., Sánchez S., Jiménez J.M., El Yousfi F., Muñoz L. (2000). Nitrogen and phosphorus removal from urban wastewater by the microalga Scenedesmus obliquus. Bioresour. Technol..

[116] Abdel-Raouf N., Al-Homaidan A.A., Ibraheem I.B.M. (2012). Microalgae and wastewater treatment. Saudi J. Biol. Sci..

[117] Zeller M.A., Hunt R., Jones A., Sharma S. (2013). Bioplastics and their thermoplastic blends from Spirulina and Chlorella microalgae. J. Appl. Polym. Sci..

[118] Rahman A., Miller C.D. (2017). Microalgae as a source of bioplastics. Algal Green Chemistry.

[119] González-Balderas R.M., Felix M., Bengoechea C., Guerrero A., Orta Ledesma M.T. (2020). Influence of mold temperature on the properties of wastewater-grown microalgae-based plastics processed by injection molding. Algal Res..

[120] Gómez-Heincke D., Martínez I., Stading M., Gallegos C., Partal P. (2017). Improvement of mechanical and water absorption properties of plant protein based bioplastics. Food Hydrocoll..

[121] Perez V., Felix M., Romero A., Guerrero A. (2016). Characterization of pea protein-based bioplastics processed by injection moulding. Food Bioprod. Process..

[122] Nadaud P., Krochta J.M. (1999). Water vapor permeability, solubility, and tensile properties of heat-denatured. Food Sci..

[123] Capezza A.J., Glad D., Özeren H.D., Newson W.R., Olsson R.T., Johansson E., Hedenqvist M.S. (2019). Novel sustainable superabsorbents: A one-pot method for functionalization of side-stream potato proteins. ACS Sustain. Chem. Eng..

[124] Ashter S.A., Ashter S.A. (2016). 7–Processing biodegradable polymers. Plastics Design Library.

[125] Lukubira S., Ogale A.A. (2013). Thermal processing and properties of bioplastic sheets derived from meat and bone meal. J. Appl. Polym. Sci..

[126] Martin-Alfonso J.E., Felix M., Romero A., Guerrero A., Martín-Alfonso J.E., Félix M., Romero A., Guerrero A. (2014). Development of new albumen based biocomposites formulations by injection moulding using chitosan as physicochemical modifier additive. Compos. Part B Eng..

[127] Gómez-Estaca J., Gavara R., Catalá R., Hernández-Muñoz P. (2016). The potential of proteins for producing food packaging materials: A review. Packag. Technol. Sci..

[128] Krochta J.M., Hernández-Izquierdo V.M. (2008). Thermoplastic processing of proteins for film formation. J. Food Sci..

[129] Budhavaram N.K., Miller J.A., Shen Y., Barone J.R. (2010). Protein substitution affects glass transition temperature and thermal stability. J. Agric. Food Chem..

[130] di Gioia L., Guilbert S. (1999). Corn protein-based thermoplastic resins: Effect of some polar and amphiphilic plasticizers. J. Agric. Food Chem..

[131] Uitto J.M., Verbeek C.J.R. (2019). The role of phase separation in determining the glass transition behaviour of thermally aggregated protein-based thermoplastics. Polym. Test..

[132] Masavang S., Roudaut G., Champion D. (2019). Identification of complex glass transition phenomena by DSC in expanded cereal-based food extrudates: Impact of plasticization by water and sucrose. J. Food Eng..

[133] Tatara R.A., Kutz M.B. (2011). 17–Compression molding. Plastics Design Library.

[134] Park C.H., Lee W.I., Advani S.G., Hsiao K.-T. (2012). 3–Compression molding in polymer matrix composites. Manufacturing Techniques for Polymer Matrix Composites (PMCs).

[135] Tatara R.A., Kutz M.B. (2017). 14–Compression molding. Plastics Design Library.

[136] Alonso-González M., Felix A., Guerrero A. (2021). Romero Effects of mould temperature on rice bran-based bioplastics obtained by injection moulding. Polymers.

[137] Otaigbe J.U., Adams D.O. (1997). Bioabsorbable soy protein plastic composites: Effect of polyphosphate fillers on water absorption and mechanical properties. J. Environ. Polym. Degrad..

[138] Tkaczyk A.H., Otaigbe J.U., Ho K.L.G. (2001). Bioabsorbable soy protein plastic composites: Effect of polyphosphate fillers on biodegradability. J. Polym. Environ..

[139] Deng R., Chen Y., Chen P., Zhang L., Liao B. (2006). Properties and biodegradability of water-resistant soy protein/poly(ε-caprolactone)/toluene-2,4-diisocyanate composites. Polym. Degrad. Stab..

[140] Huang J., Zhang L., Chen F. (2003). Effects of lignin as a filler on properties of soy protein plastics. I. Lignosulfonate. J. Appl. Polym. Sci..

[141] Nilsuwan K., Guerrero P., de la Caba K., Benjakul S., Prodpran T. (2020). Properties and application of bilayer films based on poly (lactic acid) and fish gelatin containing epigallocatechin gallate fabricated by thermo-compression molding. Food Hydrocoll..

[142] Das O., Hedenqvist M.S., Johansson E., Olsson R.T., Loho T.A., Capezza A.J., Singh Raman R.K., Holder S. (2019). An all-gluten biocomposite: Comparisons with carbon black and pine char composites. Compos. Part A Appl. Sci. Manuf..

[143] Shubhra Q.T.H., Alam A.K.M.M., Beg M.D.H. (2011). Mechanical and degradation characteristics of natural silk fiber reinforced gelatin composites. Mater. Lett..

[144] Sharma S., Luzinov I. (2013). Whey based binary bioplastics. J. Food Eng..

[145] Saenghirunwattana P., Noomhorm A., Rungsardthong V. (2014). Mechanical properties of soy protein based “green” composites reinforced with surface modified cornhusk fiber. Ind. Crops Prod..

[146] Guerrero P., de la Caba K. (2010). Thermal and mechanical properties of soy protein films processed at different pH by compression. J. Food Eng..

[147] Balaguer M.P., Gomez-Estaca J., Gavara R., Hernandez-Muñoz P. (2011). Biochemical properties of bioplastics made from wheat gliadins cross-linked with cinnamaldehyde. J. Agric. Food Chem..

[148] Zubeldía F., Ansorena M.R., Marcovich N.E. (2015). Wheat gluten films obtained by compression molding. Polym. Test..

[149] Zárate-Ramírez L.S., Martínez I., Romero A., Partal P., Guerrero A. (2011). Wheat gluten-based materials plasticised with glycerol and water by thermoplastic mixing and thermomoulding. J. Sci. Food Agric..

[150] Zárate-Ramírez L.S., Romero A., Martínez I., Bengoechea C., Partal P., Guerrero A. (2014). Effect of aldehydes on thermomechanical properties of gluten-based bioplastics. Food Bioprod. Process..

[151] Zárate-Ramírez L.S., Romero A., Bengoechea C., Partal P., Guerrero A. (2014). Thermo-mechanical and hydrophilic properties of polysaccharide/gluten-based bioplastics. Carbohydr. Polym..

[152] Martínez I., Partal P., García-Morales M., Guerrero A., Gallegos C. (2013). Development of protein-based bioplastics with antimicrobial activity by thermo-mechanical processing. J. Food Eng..

[153] Yue H.B., Cui Y.D., Shuttleworth P.S., Clark J.H. (2012). Preparation and characterisation of bioplastics made from cottonseed protein. Green Chem..

[154] Yue H.B., Cui Y.D., Yin G.Q., Jia Z.Y., Liao L.W. (2011). Environment-friendly cottonseed protein bioplastics: Preparation and properties. Adv. Mater. Res..

[155] Jerez A., Partal P., Martínez I., Gallegos C., Guerrero A., Martinez I., Gallegos C., Guerrero A. (2007). Egg white-based bioplastics developed by thermomechanical processing. J. Food Eng..

[156] Orliac O., Rouilly A., Silvestre F., Rigal L. (2002). Effects of additives on the mechanical properties, hydrophobicity and water uptake of thermo-moulded films produced from sunflower protein isolate. Polymer.

[157] De Graaf L.A. (2000). Denaturation of proteins from a non-food perspective. J. Biotechnol..

[158] Schulze C., Juraschek M., Herrmann C., Thiede S. (2017). Energy analysis of bioplastics processing. Procedia CIRP.

[159] Perez-Puyana V., Felix M., Romero A., Guerrero A. (2016). Effect of the injection moulding processing conditions on the development of pea protein-based bioplastics. J. Appl. Polym. Sci..

[160] Fernández-Espada L., Bengoechea C., Cordobés F., Guerrero A. (2016). Protein/glycerol blends and injection-molded bioplastic matrices: Soybean versus egg albumen. J. Appl. Polym. Sci..

[161] Cuadri A.A.A., Romero A., Bengoechea C., Guerrero A. (2018). The effect of carboxyl group content on water uptake capacity and tensile properties of functionalized soy protein-based superabsorbent plastics. J. Polym. Environ..

[162] Mohanty A.K., Tummala P., Liu W., Misra M., Mulukutla P.V., Drzal L.T. (2005). Injection molded biocomposites from soy protein based bioplastic and short industrial hemp fiber. J. Polym. Environ..

[163] Cuadri A.A., Bengoechea C., Romero A., Guerrero A. (2016). A natural-based polymeric hydrogel based on functionalized soy protein. Eur. Polym. J..

[164] Vaz C.M., Van Doeveren P.F.N.M., Reis R.L., Cunha A.M. (2003). Development and design of double-layer co-injection moulded soy protein based drug delivery devices. Polymer.

[165] Tummala P., Liu W., Drzal L.T., Mohanty A.K., Misra M. (2006). Influence of plasticizers on thermal and mechanical properties and morphology of soy-based bioplastics. Ind. Eng. Chem. Res..

[166] Tian H., Guo G., Xiang A., Zhong W.H. (2018). Intermolecular interactions and microstructure of glycerol-plasticized soy protein materials at molecular and nanometer levels. Polym. Test..

[167] Bourny V., Perez-Puyana V., Felix M., Romero A., Guerrero A. (2017). Evaluation of the injection moulding conditions in soy/nanoclay based composites. Eur. Polym. J..

[168] Aguilar J.M., Bengoechea C., Pérez E., Guerrero A. (2020). Effect of different polyols as plasticizers in soy based bioplastics. Ind. Crops Prod..

[169] Félix M., Lucio-Villegas A., Romero A., Guerrero A. (2016). Development of rice protein bio-based plastic materials processed by injection molding. Ind. Crops Prod..

[170] Cho S.-W., Gällstedt M., Johansson E., Hedenqvist M.S. (2011). Injection-molded nanocomposites and materials based on wheat gluten. Int. J. Biol. Macromol..

[171] Pommet M., Redl A., Morel M.-H.H., Domenek S., Guilbert S. (2003). Thermoplastic processing of protein-based bioplastics: Chemical engineering aspects of mixing, extrusion and hot molding. Macromol. Symp..

[172] Zink J., Wyrobnik T., Prinz T., Schmid M. (2016). Physical, chemical and biochemical modifications of protein-based films and coatings: An extensive review. Int. J. Mol. Sci..

[173] Robertson G.L. (2016). Food Packaging: Principles and Practice.

[174] Verbeek R.C.J., van den Berg L.E. (2010). Extrusion processing and properties of protein-based thermoplastics. Macromol. Mater. Eng..

[175] Slade L., Levine H., Ievolella J., Wang M. (1993). The glassy state phenomenon in applications for the food industry: Application of the food polymer science approach to structure--function relationships of sucrose in cookie and cracker systems. J. Sci. Food Agric..

[176] Ferry J.D. (1980). Viscoelastic Properties of Polymers.

[177] Redl A., Morel M.H., Bonicel J., Vergnes B., Guilbert S. (1999). Extrusion of wheat gluten plasticized with glycerol: Influence of process conditions on flow behavior, rheological properties, and molecular size distribution. Cereal Chem..

[178] Ullsten N.H., Cho S.-W., Spencer G., Gällstedt M., Johansson E., Hedenqvist M.S. (2009). Properties of extruded vital wheat gluten sheets with sodium hydroxide and salicylic acid. Biomacromolecules.

[179] Pommet M., Redl A., Guilbert S., Morel M.H. (2005). Intrinsic influence of various plasticizers on functional properties and reactivity of wheat gluten thermoplastic materials. J. Cereal Sci..

[180] Ullsten N.H., Gällstedt M., Spencer G.M., Johansson E., Marttila S., Ignell R., Hedenqvist M.S. (2010). Extruded high quality materials from wheat gluten. Polym. Renew. Resour..

[181] Pietsch V.L., Werner R., Karbstein H.P., Emin M.A. (2019). High moisture extrusion of wheat gluten: Relationship between process parameters, protein polymerization, and final product characteristics. J. Food Eng..

[182] Pietsch V.L., Schöffel F., Rädle M., Karbstein H.P., Emin M.A. (2019). High moisture extrusion of wheat gluten: Modeling of the polymerization behavior in the screw section of the extrusion process. J. Food Eng..

[183] Bengoechea C., Arrachid A., Guerrero A., Hill S.E., Mitchell J.R. (2007). Relationship between the glass transition temperature and the melt flow behavior for gluten, casein and soya. J. Cereal Sci..

[184] Arêas J.A.G. (1992). Extrusion of food proteins. Crit. Rev. Food Sci. Nutr..

[185] Felix M., Martinez I., Romero A., Partal P., Guerrero A. (2018). Effect of pH and nanoclay content on the morphology and physicochemical properties of soy protein/montmorillonite nanocomposite obtained by extrusion. Compos. Part B Eng..

[186] Liu W.J., Misra M., Askeland P., Drzal L.T., Mohanty A.K. (2005). “Green” composites from soy based plastic and pineapple leaf fiber: Fabrication and properties evaluation. Polymer.

[187] Liu B., Jiang L., Zhang J. (2010). Development of soy protein/poly (lactic acid) bioplastics. GPEC 2010, Proceedings of the Global Plastics Environmental Conference 2010: Sustainability and Recycling, Orlando, FL, USA, 8–11 March 2010.

[188] Liu W., Mohanty A.K., Askeland P., Drzal L.T., Misra M. (2004). Influence of fiber surface treatment on properties of Indian grass fiber reinforced soy protein based biocomposites. Polymer.

[189] Zhang J., Mungara P., Jane J. (2001). Mechanical and thermal properties of extruded soy protein sheets. Polymer.

[190] Saptarshi S.M., Zhou D.C. (2019). Basics of 3D Printing.

[191] Chia H.N., Wu B.M. (2015). Recent advances in 3D printing of biomaterials. J. Biol. Eng..

[192] Bose S., Vahabzadeh S., Bandyopadhyay A. (2013). Bone tissue engineering using 3D printing. Mater. Today.

[193] Krishnadoss V., Kanjilal B., Hesketh A., Miller C., Mugweru A., Akbard M., Khademhosseini A., leijten J., Noshadi I. (2021). In situ 3D printing of implantable energy storage devices. Chem. Eng. J..

[194] Zeng L., Li P., Yao Y., Niu B., Niu S., Xu B. (2020). Recent progresses of 3D printing technologies for structural energy storage devices. Mater. Today Nano.

[195] Sun J., Zhou W., Huang D. (2018). 3D Printing of Food.

[196] Kim H.W., Bae H., Park H.J. (2017). Classification of the printability of selected food for 3D printing: Development of an assessment method using hydrocolloids as reference material. J. Food Eng..

[197] Oyinloye T.M., Yoon W.B. (2021). Stability of 3D printing using a mixture of pea protein and alginate: Precision and application of additive layer manufacturing simulation approach for stress distribution. J. Food Eng..

[198] Álvarez-Castillo E., Oliveira S., Bengoechea C., Sousa I., Raymundo A., Guerrero A. (2021). A rheological approach to 3D printing of plasma protein based doughs. J. Food Eng..

[199] Chen J., Mu T., Goffin D., Blecker C., Richard G., Richel A., Haubruge E. (2019). Application of soy protein isolate and hydrocolloids based mixtures as promising food material in 3D food printing. J. Food Eng..

[200] Liu Y., Yu Y., Liu C., Regenstein J.M., Liu X., Zhou P. (2019). Rheological and mechanical behavior of milk protein composite gel for extrusion-based 3D food printing. LWT.

[201] Liu Y., Liu D., Wei G., Ma Y., Bhandari B., Zhou P. (2018). 3D printed milk protein food simulant: Improving the printing performance of milk protein concentration by incorporating whey protein isolate. Innov. Food Sci. Emerg. Technol..

[202] Farris S., Introzzi L., Piergiovanni L. (2009). Evaluation of a bio-coating as a solution to improve barrier, friction and optical properties of plastic films. Packag. Technol. Sci..

[203] Muriel-Galet V., Cerisuelo J.P., López-Carballo G., Aucejo S., Gavara R., Hernández-Muñoz P. (2013). Evaluation of EVOH-coated PP films with oregano essential oil and citral to improve the shelf-life of packaged salad. Food Control.

[204] Wihodo M., Moraru C.I. (2013). Physical and chemical methods used to enhance the structure and mechanical properties of protein films: A review. J. Food Eng..

[205] Bernard C., Christian A., Jean-Louis C., Stéphane G. (2018). Edible packaging films based on fish myofibrillar proteins: Formulation and functional properties. J. Food Sci..

[206] Guilbert S., Morel M.-H., Gontard N., Cuq B. (2006). Protein-based plastics and composites as smart green materials. ACS Symp. Ser..

[207] Lagrain B., Goderis B., Brijs K., Delcour J.A. (2010). Molecular basis of processing wheat gluten toward biobased materials. Biomacromolecules.

[208] Newson W.R. (2012). Protein Based Plastics from the Residuals of Industrial Oil Crops.

[209] Mangavel C., Barbot J., Bervas E., Linossier L., Feys M., Gueèguen J., Popineau Y. (2002). Influence of prolamin composition on mechanical properties of cast wheat gluten films. J. Cereal Sci..

[210] Hernández-Muñoz P., Kanavouras A., Ng P.K.W., Gavara R. (2003). Development and characterization of biodegradable films made from wheat gluten protein fractions. J. Agric. Food Chem..

[211] Hernández-Muñoz P., Villalobos R., Chiralt A. (2004). Effect of thermal treatments on functional properties of edible films made from wheat gluten fractions. Food Hydrocoll..

[212] Balaguer M.P., Gómez-Estaca J., Gavara R., Hernandez-Munoz P. (2011). Functional properties of bioplastics made from wheat gliadins modified with cinnamaldehyde. J. Agric. Food Chem..

[213] Pommet M., Redl A., Morel M.-H.H., Guilbert S. (2003). Study of wheat gluten plasticization with fatty acids. Polymer.

[214] Gontard N., Guilbert S., Cuq J.-L. (1992). Edible wheat gluten films: Influence of the main process variables on film properties using response surface methodology. J. Food Sci..

[215] Gontard N., Guilbert S. (1994). Bio-packaging: Technology and properties of edible and/or biodegradable material of agricultural origin. Food Packaging and Preservation.

[216] Lim S.-T., Jane J.-L., Rajagopalan S., Seib P.A. (1992). Effect of starch granule size on physical properties of starch-filled polyethylene film. Biotechnol. Prog..

[217] Cuq B., Gontard N., Cuq J.-L., Guilbert S. (1996). Stability of myofibrillar protein-based biopackagings during storage. LWT Food Sci. Technol..

[218] Tunc S., Angellier H., Cahyana Y., Chalier P., Gontard N., Gastaldi E. (2007). Functional properties of wheat gluten/montmorillonite nanocomposite films processed by casting. J. Memb. Sci..

[219] Kayserilioğlu B.Ş., Bakir U., Yilmaz L., Akkaş N. (2003). Use of xylan, an agricultural by-product, in wheat gluten based biodegradable films: Mechanical, solubility and water vapor transfer rate properties. Bioresour. Technol..

[220] Micard V., Morel M.H., Bonicel J., Guilbert S. (2001). Thermal properties of raw and processed wheat gluten in relation with protein aggregation. Polymer.

[221] Guilbert S., Gontard N., Cuq B. (1995). Technology and applications of edible protective films. Packag. Technol. Sci..

[222] Heralp T.J., Gnanasambandam R., McGuire B.H., Hachmeister K.A. (1995). Degradable wheat gluten Films: Preparation, properties and applications. J. Food Sci..

[223] Gennadios A., Brandenburg A.H., Weller C.L., Testin R.F. (1993). Effect of pH on properties of wheat gluten and soy protein isolate films. J. Agric. Food Chem..

[224] Gontard N., Guilbert S.S., Cuq J.-L.J.-L., Nathalie G., Stéphane G., Jean-Louis C.U.Q., Gontard N., Guilbert S.S., Cuq J.-L.J.-L. (1993). Water and glycerol as plasticizers affect mechanical and water vapor barrier properties of an edible wheat gluten film. J. Food Sci..

[225] Gontard N., Duchez C., CUQ J.-L., Guilbert S. (1994). Edible composite films of wheat gluten and lipids: Water vapour permeability and other physical properties. Int. J. Food Sci. Technol..

[226] Gennadios A., Weller C.L. (1990). Edible films and coatings from wheat and corn proteins. Food Technol..

[227] Ghanbarzodeh B., Oromiehie A.R., Musavi M., Falcone P.M., D.-Jomeh Z.E., Rad E.R. (2007). Study of mechanical properties, oxygen permeability and AFM topography of zein films plasticized by polyols. Packag. Technol. Sci..

[228] Martin D.N. (1948). Zein-containing plastic composition. Law Contemp. Probl..

[229] Lai H.-M., Padua G.W. (1997). Properties and microstructure of plasticized zein films. Cereal Chem..

[230] Kim K.M., Weller C.L., Hanna M.A., Gennadios A. (2002). Heat curing of soy protein films at selected temperatures and pressures. LWT Food Sci. Technol..

[231] Rhim J.W., Gennadios A., Handa A., Weller C.L., Hanna M.A. (2000). Solubility, tensile, and color properties of modified soy protein isolate films. J. Agric. Food Chem..

[232] Zhou J.J., Wang S.Y., Gunasekaran S. (2009). Preparation and characterization of whey protein film incorporated with TiO2 nanoparticles. J. Food Sci..

[233] Sothornvit R., Krochta J.M. (2000). Water vapor permeability and solubility of films from hydrolyzed whey protein. J. Food Sci..

[234] Avena-Bustillos R.J., Krochta J.M. (1993). Water vapor permeability of caseinate-based edible films as affected by pH, calcium crosslinking and lipid content. J. Food Sci..

[235] Sohail S.S., Wang B., Biswas M.A.S., Oh J.H. (2006). Physical, morphological, and barrier properties of edible casein films with wax applications. J. Food Sci..

[236] Sothornvit R., Krochta J.M. (2000). Plasticizer effect on oxygen permeability of β-lactoglobulin films. J. Agric. Food Chem..

[237] Acquah C., Zhang Y., Dubé M.A., Udenigwe C.C. (2020). Formation and characterization of protein-based films from yellow pea (Pisum sativum) protein isolate and concentrate for edible applications. Curr. Res. Food Sci..

[238] Choi W.-S., Han J.H. (2001). Physical and mechanical properties of pea-protein-based edible films. J. Food Sci..

[239] Choi W.S., Han J.H. (2002). Film-forming mechanism and heat denaturation effects on the physical and chemical properties of pea-protein-isolate edible films. J. Food Sci..

[240] Kowalczyk D., Gustaw W., Świeca Michałand Baraniak B. (2014). A study on the mechanical properties of pea protein isolate films. J. Food Process. Preserv..

[241] Araújo C.S., Rodrigues A.M.C., Peixoto Joele M.R.S., Araújo E.A.F., Lourenço L.F.H. (2018). Optmizing process parameters to obtain a bioplastic using proteins from fish byproducts through the response surface methodology. Food Packag. Shelf Life.

[242] Cuq B., Gontard N., Guilbert S. (1997). Thermoplastic properties of fish myofibrillar proteins: Application to biopackaging fabrication. Polymer.

[243] Ansari F.A., Ravindran B., Gupta S.K., Nasr M., Rawat I., Bux F. (2019). Techno-economic estimation of wastewater phycoremediation and environmental benefits using Scenedesmus obliquus microalgae. J. Environ. Manag..

[244] Ahmad M., Hani N.M., Nirmal N.P., Fazial F.F., Mohtar N.F., Romli S.R. (2015). Optical and thermo-mechanical properties of composite films based on fish gelatin/rice flour fabricated by casting technique. Prog. Org. Coat..

[245] Fabra M.J., López-Rubio A., Lagaron J.M. (2016). Use of the electrohydrodynamic process to develop active/bioactive bilayer films for food packaging applications. Food Hydrocoll..

[246] Huang Z.M., Zhang Y.Z., Ramakrishna S., Lim C.T. (2004). Electrospinning and mechanical characterization of gelatin nanofibers. Polymer.

[247] Bhushani J.A., Anandharamakrishnan C. (2014). Electrospinning and electrospraying techniques: Potential food based applications. Trends Food Sci. Technol..

[248] Schiffman J.D., Schauer C.L. (2008). A review: Electrospinning of biopolymer nanofibers and their applications. Polym. Rev..

[249] Wang X., Hsiao B.S. (2016). Electrospun nanofiber membranes. Curr. Opin. Chem. Eng..

[250] Ramakrishna S. (2005). An Introduction to Electrospinning and Nanofibers.

[251] Mendes A.C., Stephansen K., Chronakis I.S. (2017). Electrospinning of food proteins and polysaccharides. Food Hydrocoll..

[252] Aviss K.J., Gough J.E., Downes S. (2010). Aligned electrospun polymer fibres for skeletal muscle regeneration. Eur. Cells Mater..

[253] Pan H., Li L., Hu L., Cui X. (2006). Continuous aligned polymer fibers produced by a modified electrospinning method. Polymer.

[254] Teo W.E., Ramakrishna S. (2006). A review on electrospinning design and nanofibre assemblies. Nanotechnology.

[255] Kriegel C., Arrechi A., Kit K., McClements D.J., Weiss J. (2008). Fabrication, functionalization, and application of electrospun biopolymer nanofibers. Crit. Rev. Food Sci. Nutr..

[256] Dror Y., Ziv T., Makarov V., Wolf H., Admon A., Zussman E. (2008). Nanofibers made of globular proteins. Biomacromolecules.

[257] Regev O., Khalfin R., Zussman E., Cohen Y. (2010). About the albumin structure in solution and related electro-spinnability issues. Int. J. Biol. Macromol..

[258] Wongsasulak S., Kit K.M., McClements D.J., Yoovidhya T., Weiss J. (2007). The effect of solution properties on the morphology of ultrafine electrospun egg albumen-PEO composite fibers. Polymer.

[259] Huang G.P., Shanmugasundaram S., Masih P., Pandya D., Amara S., Collins G., Arinzeh T.L. (2015). An investigation of common crosslinking agents on the stability of electrospun collagen scaffolds. J. Biomed. Mater. Res. Part A.

[260] Gautam S., Dinda A.K., Mishra N.C. (2013). Fabrication and characterization of PCL/gelatin composite nanofibrous scaffold for tissue engineering applications by electrospinning method. Mater. Sci. Eng. C Mater. Biol. Appl..

[261] Elamparithi A., Punnoose A.M., Kuruvilla S. (2016). Electrospun type 1 collagen matrices preserving native ultrastructure using benign binary solvent for cardiac tissue engineering. Artif. Cells Nanomed. Biotechnol..

[262] Song F., Tang D.-L., Wang X.-L., Wang Y.-Z. (2011). Biodegradable soy protein isolate-based materials: A review. Biomacromolecules.

[263] Wongsasulak S., Patapeejumruswong M., Weiss J., Supaphol P., Yoovidhya T. (2010). Electrospinning of food-grade nanofibers from cellulose acetate and egg albumen blends. J. Food Eng..

[264] Lang G., Jokisch S., Scheibel T. (2013). Air filter devices including nonwoven meshes of electrospun recombinant spider silk proteins. J. Vis. Exp..

[265] Aman mohammadi M., Ramazani S., Rostami M., Raeisi M., Tabibiazar M., Ghorbani M. (2019). Fabrication of food-grade nanofibers of whey protein Isolate–Guar gum using the electrospinning method. Food Hydrocoll..

[266] Callister W.D. (2007). Materials Science and Engineering: An Introduction.

[267] Garavand F., Rouhi M., Razavi S.H., Cacciotti I., Mohammadi R. (2017). Improving the integrity of natural biopolymer films used in food packaging by crosslinking approach: A review. Int. J. Biol. Macromol..

[268] Felix M., Perez-Puyana V., Romero A., Guerrero A. (2017). Development of protein-based bioplastics modified with different additives. J. Appl. Polym. Sci..

[269] Uchikawa H. (2001). Specialized techniques. Handb. Anal. Tech. Concr. Sci. Technol..

[270] Barnes H.A. (2000). A Handbook of Elementary Rheology.

[271] Banks H.T., Hu S., Kenz Z.R. (2011). A brief review of elasticity and viscoelasticity for solids. Adv. Appl. Math. Mech..

[272] Gomez-Martinez D., Partal P., Martinez I., Gallegos C. (2009). Rheological behaviour and physical properties of controlled-release gluten-based bioplastics. Bioresour. Technol..

[273] Sun S., Song Y., Zheng Q. (2008). Morphology and mechanical properties of thermo-molded bioplastics based on glycerol-plasticized wheat gliadins. J. Cereal Sci..

[274] Felix M., Romero A., Cordobes F., Guerrero A. (2015). Development of crayfish bio-based plastic materials processed by small-scale injection moulding. J. Sci. Food Agric..

[275] Domenek S., Feuilloley P., Gratraud J., Morel M.-H., Guilbert S. (2004). Biodegradability of wheat gluten based bioplastics. Chemosphere.

[276] Ghanbarzadeh B., Oromiehie A.R., Musavi M., D.-Jomeh Z.E., Rad E.R., Milani J. (2006). Effect of plasticizing sugars on rheological and thermal properties of zein resins and mechanical properties of zein films. Food Res. Int..

[277] Félix M., Romero A., Martín-Alfonso J.E., Guerrero A. (2015). Development of crayfish protein-PCL biocomposite material processed by injection moulding. Compos. Part B Eng..

[278] Felix M., Carpintero V., Romero A., Guerrero A. (2016). Influence of sorbitol on mechanical and physico-chemical properties of soy protein-based bioplastics processed by injection molding. Polimeros.

[279] Martucci J.F., Ruseckaite R.A., Vázquez A. (2006). Creep of glutaraldehyde-crosslinked gelatin films. Mater. Sci. Eng. A.

[280] Reddy D.J.P., Rajulu A.V., Arumugam V., Naresh M.D., Muthukrishnan M. (2009). Effects of resorcinol on the mechanical properties of soy protein isolate films. J. Plast. Film Sheeting.

[281] Sochava I.V., Smirnova O.I. (1993). Heat capacity of hydrated and dehydrated globular proteins. Denaturation increment of heat capacity. Food Hydrocoll..

[282] Chen P., Zhang L., Cao F. (2005). Effects of moisture on glass transition and microstructure of glycerol-plasticized soy protein. Macromol. Biosci..

[283] Ricci L., Umiltà E., Righetti M.C., Messina T., Zurlini C., Montanari A., Bronco S., Bertoldo M. (2018). On the thermal behavior of protein isolated from different legumes investigated by DSC and TGA. J. Sci. Food Agric..

[284] López Rocha C.J., Álvarez-Castillo E., Estrada Yáñez M.R., Bengoechea C., Guerrero A., Orta Ledesma M.T. (2020). Development of bioplastics from a microalgae consortium from wastewater. J. Environ. Manag..

[285] Li F., Liu T., Gu W., Gao Q., Li J., Shi S.Q. (2021). Bioinspired super-tough and multifunctional soy protein-based material via a facile approach. Chem. Eng. J..

[286] Sun S., Song Y., Zheng Q. (2007). Morphologies and properties of thermo-molded biodegradable plastics based on glycerol-plasticized wheat gluten. Food Hydrocoll..

[287] Guerrero A., Carmona J., Martinez I., Cordobes F., Partal P. (2004). Effect of pH and added electrolyte on the thermal-induced transitions of egg yolk. Rheol. Acta.

[288] Soukoulis C., Behboudi-Jobbehdar S., Macnaughtan W., Parmenter C., Fisk I.D. (2017). Stability of Lactobacillus rhamnosus GG incorporated in edible films: Impact of anionic biopolymers and whey protein concentrate. Food Hydrocoll..

[289] Jones A., Zeller M.A., Sharma S. (2013). Thermal, mechanical, and moisture absorption properties of egg white protein bioplastics with natural rubber and glycerol. Prog. Biomater..

[290] Jerez A., Partal P., Martinez I., Gallegos C., Guerrero A. (2007). Protein-based bioplastics: Effect of thermo-mechanical processing. Rheol. Acta.

[291] Gracia-Fernández C.A., Gómez-Barreiro S., López-Beceiro J., Tarrío Saavedra J., Naya S., Artiaga R. (2010). Comparative study of the dynamic glass transition temperature by DMA and TMDSC. Polym. Test..

[292] Álvarez-Castillo E., Pelagio M.J., Bengoechea C., Guerrero A. (2021). Plasma based superabsorbent materials modulated through chemical cross-linking. J. Environ. Chem. Eng..

[293] Álvarez-Castillo E., Caballero G., Guerrero A., Bengoechea C. (2021). Effect of formulation and pressure on injection moulded soy protein-based plastics. J. Polym. Environ..

[294] Oymaci P., Altinkaya S.A. (2016). Improvement of barrier and mechanical properties of whey protein isolate based food packaging films by incorporation of zein nanoparticles as a novel bionanocomposite. Food Hydrocoll..

[295] Thammahiwes S., Riyajan S.-A., Kaewtatip K. (2017). Preparation and properties of wheat gluten based bioplastics with fish scale. J. Cereal Sci..

[296] Sukyai P., Anongjanya P., Bunyahwuthakul N., Kongsin K., Harnkarnsujarit N., Sukatta U., Sothornvit R., Chollakup R. (2018). Effect of cellulose nanocrystals from sugarcane bagasse on whey protein isolate-based films. Food Res. Int..

[297] Diañez I., Martínez I., Partal P. (2016). Synergistic effect of combined nanoparticles to elaborate exfoliated egg-white protein-based nanobiocomposites. Compos. Part B Eng..

[298] Perotto G., Ceseracciu L., Simonutti R., Paul U., Guzman-Puyol S., Tran T.-N., Bayer I., Athanassiou A. (2018). Bioplastics from vegetable waste via an eco-friendly water-based process. Green Chem..

[299] González-Gutiérrez J., Partal P., García-Morales M., Gallegos C. (2011). Effect of processing on the viscoelastic, tensile and optical properties of albumen/starch-based bioplastics. Carbohydr. Polym..

[300] Gounga M.E., Xu S.-Y., Wang Z. (2007). Whey protein isolate-based edible films as affected by protein concentration, glycerol ratio and pullulan addition in film formation. J. Food Eng..

[301] Karmee S.K., Lin C.S.K. (2014). Valorisation of food waste to biofuel: Current trends and technological challenges. Sustain. Chem. Process..

[302] Secchi G. (2008). Role of protein in cosmetics. Clin. Dermatol..

[303] Bradley E.L., Castle L., Chaudhry Q. (2011). Applications of nanomaterials in food packaging with a consideration of opportunities for developing countries. Trends Food Sci. Technol..

[304] Chen H., Wang J., Cheng Y., Wang C., Liu H., Bian H., Pan Y., Sun J., Han W. (2019). Application of protein-based films and coatings for food packaging: A review. Polymers.

[305] Hong L.G., Yuhana N.Y., Zawawi E.Z.E. (2021). Review of bioplastics as food packaging materials. AIMS Mater. Sci..

[306] Mihalca V., Kerezsi A.D., Weber A., Gruber-Traub C., Schmucker J., Vodnar D.C., Dulf F.V., Socaci S.A., Fărcaș A., Mureșan C.I. (2021). Protein-based films and coatings for food industry applications. Polymers.

[307] Moschopoulou E., Moatsou G., Syrokou M.K., Paramithiotis S., Drosinos E.H., Galanakis C.M. (2019). 1–Food quality changes during shelf life. Food Quality and Shelf Life.

[308] Cavallo J.A., Strumia M.C., Gomez C.G. (2014). Preparation of a milk spoilage indicator adsorbed to a modified polypropylene film as an attempt to build a smart packaging. J. Food Eng..

[309] Nychas G.-J.E., Tassou C.C., Batt C.A., Tortorello M.L. (2014). Preservatives. Traditional preservatives–Oils and spices. Encyclopedia of Food Microbiology.

[310] Balasubramaniam V.M., Chinnan M.S., Erickson M.C., Hung Y.-C. (1997). Role of packaging in quality preservation of frozen foods. Quality in Frozen Food.

[311] Park H.J., Chinnan M.S., Shewfelt R.L. (1994). Edible coating effects on storage life and quality of tomatoes. J. Food Sci..

[312] Park H.J., Jo K.H. (1996). Edible coating effect on Korean “Fuji” apples and “shingo” pear. IFT Annual Meeting. Book of Abstracts, Proceedings of the IFT Annual Meeting, New Orleans, LA, USA, 22–26 June 1996.

[313] Ünalan İ.U., Arcan I., Korel F., Yemenicioğlu A. (2013). Application of active zein-based films with controlled release properties to control Listeria monocytogenes growth and lipid oxidation in fresh Kashar cheese. Innov. Food Sci. Emerg. Technol..

[314] Han J., Bourgeois S., Lacroix M. (2009). Protein-based coatings on peanut to minimise oil migration. Food Chem..

[315] Etxabide A., Uranga J., Guerrero P., de la Caba K. (2017). Development of active gelatin films by means of valorisation of food processing waste: A review. Food Hydrocoll..

[316] Poverenov E., Zaitsev Y., Arnon H., Granit R., Alkalai-Tuvia S., Perzelan Y., Weinberg T., Fallik E. (2014). Effects of a composite chitosan--gelatin edible coating on postharvest quality and storability of red bell peppers. Postharvest Biol. Technol..

[317] Ramos M., Valdes A., Beltran A., Garrigós M.C. (2016). Gelatin-based films and coatings for food packaging applications. Coatings.

[318] Nagarajan M., Benjakul S., Prodpran T., Songtipya P. (2015). Effects of bio-nanocomposite films from tilapia and squid skin gelatins incorporated with ethanolic extract from coconut husk on storage stability of mackerel meat powder. Food Packag. Shelf Life.

[319] Yangılar F. (2015). Chitosan/whey Protein (CWP) edible films efficiency for controlling mould growth and on microbiological, chemical and sensory properties during storage of Göbek Kashar cheese. Korean J. Food Sci. Anim. Resour..

[320] Wagh Y.R., Pushpadass H.A., Emerald F.M.E., Nath B.S. (2014). Preparation and characterization of milk protein films and their application for packaging of Cheddar cheese. J. Food Sci. Technol..

[321] Zinoviadou K.G., Koutsoumanis K.P., Biliaderis C.G. (2010). Physical and thermo-mechanical properties of whey protein isolate films containing antimicrobials, and their effect against spoilage flora of fresh beef. Food Hydrocoll..

[322] Calva-Estrada S.J., Jiménez-Fernández M., Lugo-Cervantes E. (2019). Protein-based films: Advances in the development of biomaterials applicable to food packaging. Food Eng. Rev..

[323] Aguilera Barraza F.A., León R.A.Q., Álvarez P.X.L. (2015). Kinetics of protein and textural changes in Atlantic salmon under frozen storage. Food Chem..

[324] Rodriguez-Turienzo L., Cobos A., Moreno V., Caride A., Vieites J.M., Diaz O. (2011). Whey protein-based coatings on frozen Atlantic salmon (Salmo salar): Influence of the plasticiser and the moment of coating on quality preservation. Food Chem..

[325] Chen Y., Tan H. (2006). Crosslinked carboxymethylchitosan-g-poly (acrylic acid) copolymer as a novel superabsorbent polymer. Carbohydr. Res..

[326] Commission E. (2009). EU Guidance to the Commission Regulation (EC) No 450/2009 of 29 May 2009 on active and intelligent materials and articles intended to come into the contact with food (version 1.0). Off. J. Eur. Union.

[327] Realini C.E., Marcos B. (2014). Active and intelligent packaging systems for a modern society. Meat Sci..

[328] Batista R.A., Espitia P.J.P., de Souza Siqueira Quintans J., Freitas M.M., Cerqueira M.Â., Teixeira J.A., Cardoso J.C. (2019). Hydrogel as an alternative structure for food packaging systems. Carbohydr. Polym..

[329] Weng W., Zheng H. (2015). Effect of transglutaminase on properties of tilapia scale gelatin films incorporated with soy protein isolate. Food Chem..

[330] Han J.H., Han J.H. (2005). 6–Antimicrobial packaging systems. Innovations in Food Packaging.

[331] Appendini P., Hotchkiss J.H. (2002). Review of antimicrobial food packaging. Innov. Food Sci. Emerg. Technol..

[332] Seydim A.C., Sarikus G. (2006). Antimicrobial activity of whey protein based edible films incorporated with oregano, rosemary and garlic essential oils. Food Res. Int..

[333] Padgett T., Han I.Y., Dawson P.L. (1998). Incorporation of food-grade antimicrobial compounds into biodegradable packaging films. J. Food Prot..

[334] Redl A., Gontard N., Guilbert S. (1996). Determination of sorbic acid diffusivity in edible wheat gluten and lipid based films. J. Food Sci..

[335] López-Caballero M.E., Gómez-Guillén M.C., Pérez-Mateos M., Montero P. (2005). A chitosan–gelatin blend as a coating for fish patties. Food Hydrocoll..

[336] Domínguez R., Barba F.J., Gómez B., Putnik P., Bursać Kovačević D., Pateiro M., Santos E.M., Lorenzo J.M. (2018). Active packaging films with natural antioxidants to be used in meat industry: A review. Food Res. Int..

[337] Delgado-Adámez J., Bote E., Parra-Testal V., Martín M.J., Ramírez R. (2016). Effect of the olive leaf extracts in vitro and in active packaging of sliced iberian pork loin. Packag. Technol. Sci..

[338] Guillen M.D., Goicoechea E. (2008). Formation of oxygenated α, β-unsaturated aldehydes and other toxic compounds in sunflower oil oxidation at room temperature in closed receptacles. Food Chem..

[339] Lorenzo J.M., Batlle R., Gómez M. (2014). Extension of the shelf-life of foal meat with two antioxidant active packaging systems. LWT Food Sci. Technol..

[340] Maryam Adilah Z.A., Nur Hanani Z.A. (2016). Active packaging of fish gelatin films with Morinda citrifolia oil. Food Biosci..

[341] Colín-Chávez C., Vicente-Ramírez E.B., Soto-Valdez H., Peralta E., Auras R. (2014). The release of carotenoids from a light-protected antioxidant active packaging designed to improve the stability of soybean oil. Food Bioprocess Technol..

[342] Tian F., Decker E.A., Goddard J.M. (2013). Controlling lipid oxidation of food by active packaging technologies. Food Funct..

[343] Vanden Braber N.L., Di Giorgio L., Aminahuel C.A., Díaz Vergara L.I., Martín Costa A.O., Montenegro M.A., Mauri A.N. (2021). Antifungal whey protein films activated with low quantities of water soluble chitosan. Food Hydrocoll..

[344] Omidian H., Zohuriaan-Mehr M.J., Kabiri K., Shah K. (2004). Polymer chemistry attractiveness: Synthesis and swelling studies of gluttonous hydrogels in the advanced academic laboratory. J. Polym. Mater..

[345] Choudhary B., Paul S.R., Nayak S.K., Qureshi D., Pal K., Pal K., Banerjee I. (2018). Synthesis and biomedical applications of filled hydrogels. Polymeric Gels.

[346] Zohuriaan-Mehr M.J., Kabiri K. (2008). Superabsorbent polymer materials: A review. Iran. Polym. J..

[347] Mignon A., De Belie N., Dubruel P., Van Vlierberghe S. (2019). Superabsorbent polymers: A review on the characteristics and applications of synthetic, polysaccharide-based, semi-synthetic and ‘smart’ derivatives. Eur. Polym. J..

[348] Bidgoli H., Zamani A., Taherzadeh M.J. (2010). Effect of carboxymethylation conditions on the water-binding capacity of chitosan-based superabsorbents. Carbohydr. Res..

[349] Elbarbary A.M., El-Rehim H.A.A., El-Sawy N.M., Hegazy E.S.A., Soliman E.S.A. (2017). Radiation induced crosslinking of polyacrylamide incorporated low molecular weights natural polymers for possible use in the agricultural applications. Carbohydr. Polym..

[350] Thombare N., Mishra S., Siddiqui M.Z., Jha U., Singh D., Mahajan G.R. (2018). Design and development of guar gum based novel, superabsorbent and moisture retaining hydrogels for agricultural applications. Carbohydr. Polym..

[351] Felix M., Martínez I., Aguilar J.M., Guerrero A. (2018). Development of biocomposite superabsorbent nanomaterials: Effect of processing technique. J. Polym. Environ..

[352] Sadeghi M., Hosseinzadeh H. (2010). Swelling Behaviour of a novel protein-based super absorbent hydrogel composed of poly (methacrylic acid) and collagen. Asian, J. Chem..

[353] Zhao Y., He M., Zhao L., Wang S., Li Y., Gan L., Li M., Xu L., Chang P.R., Anderson D.P. (2016). Epichlorohydrin-cross-linked hydroxyethyl cellulose/soy protein isolate composite films as biocompatible and biodegradable implants for tissue engineering. ACS Appl. Mater. Interfaces.

[354] Ali A., Ahmed S. (2018). Recent advances in edible polymer based hydrogels as a sustainable alternative to conventional polymers. J. Agric. Food Chem..

[355] Zohuriaan-Mehr M.J., Omidian H., Doroudiani S., Kabiri K. (2010). Advances in non-hygienic applications of superabsorbent hydrogel materials. J. Mater. Sci..

[356] Pearlstein L. (1998). Absorbent Packaging for Food Products. U.S. Patent.

[357] Etchells M.D., Versteylen S. (2010). Absorbent Food Pad and Method of Using Same. U.S. Patent.

[358] Pawlowski T.D., Ticknor W.G. (1990). Absorbent Insert for Food Packages. U.S. Patent.

[359] Ni N., Zhang D., Dumont M.-J. (2018). Synthesis and characterization of zein-based superabsorbent hydrogels and their potential as heavy metal ion chelators. Polym. Bull..

[360] Gunasekaran S., Xiao L., Ould Eleya M.M. (2006). Whey protein concentrate hydrogels as bioactive carriers. J. Appl. Polym. Sci..

[361] Song F., Zhang L.-M., Shi J.-F., Li N.-N. (2010). Novel casein hydrogels: Formation, structure and controlled drug release. Colloids Surf. B Biointerfaces.

[362] Song F., Zhang L.-M., Yang C., Yan L. (2009). Genipin-crosslinked casein hydrogels for controlled drug delivery. Int. J. Pharm..

[363] Shi W., Dumont M.-J.J., Ly E.B. (2014). Synthesis and properties of canola protein-based superabsorbent hydrogels. Eur. Polym. J..

[364] Samsalee N., Sothornvit R. (2019). Development and characterization of porcine plasma protein-chitosan blended films. Food Packag. Shelf Life.

[365] Nuthong P., Benjakul S., Prodpran T. (2009). Effect of some factors and pretreatment on the properties of porcine plasma protein-based films. LWT Food Sci. Technol..

[366] Nuthong P., Benjakul S., Prodpran T. (2009). Effect of phenolic compounds on the properties of porcine plasma protein-based film. Food Hydrocoll..

[367] Amonpattaratkit P., Khunmanee S., Kim D.H., Park H. (2017). Synthesis and characterization of gelatin-based crosslinkers for the fabrication of superabsorbent hydrogels. Materials.

[368] Berton S.B.R., de Jesus G.A.M., Sabino R.M., Monteiro J.P., Venter S.A.S., Bruschi M.L., Popat K.C., Matsushita M., Martins A.F., Bonafé E.G. (2020). Properties of a commercial κ-carrageenan food ingredient and its durable superabsorbent hydrogels. Carbohydr. Res..

[369] Yadav M., Rhee K.Y. (2012). Superabsorbent nanocomposite (alginate-g-PAMPS/MMT): Synthesis, characterization and swelling behavior. Carbohydr. Polym..

[370] Capezza A.J., Cui Y., Numata K., Lundman M., Newson W.R., Olsson R.T., Johansson E., Hedenqvist M.S. (2020). High capacity functionalized protein superabsorbents from an agricultural co-product: A cradle-to-cradle approach. Adv. Sustain. Syst..

[371] Iles A., Martin A.N. (2013). Expanding bioplastics production: Sustainable business innovation in the chemical industry. J. Clean. Prod..

[372] Da Costa J.P., Nunes A.R., Santos P.S.M., Girão A.V., Duarte A.C., Rocha-Santos T. (2018). Degradation of polyethylene microplastics in seawater: Insights into the environmental degradation of polymers. J. Environ. Sci. Heal. Part A.

[373] Ariza-Tarazona M.C., Villarreal-Chiu J.F., Barbieri V., Siligardi C., Cedillo-González E.I. (2019). New strategy for microplastic degradation: Green photocatalysis using a protein-based porous N-TiO2 semiconductor. Ceram. Int..

[374] Fendall L.S., Sewell M.A. (2009). Contributing to marine pollution by washing your face: Microplastics in facial cleansers. Mar. Pollut. Bull..

[375] Law K.L., Thompson R.C. (2014). Microplastics in the seas. Science.

[376] Behera S., Mahanwar P.A. (2020). Superabsorbent polymers in agriculture and other applications: A review. Polym. Technol. Mater..

[377] Jiménez-Rosado M., Perez-Puyana V., Sánchez-Cid P., Guerrero A., Romero A. (2021). Incorporation of ZnO nanoparticles into soy protein-based bioplastics to improve their functional properties. Polymers.

[378] Sayyari M., Ghanbari F. (2012). Effects of super absorbent polymer A200 on the growth, yield and some physiological responses in sweet pepper (Capsicum annuum L.) under various irrigation regimes. Int. J. Agric. Food Res..

[379] Ni B., Liu M., Lü S. (2009). Multifunctional slow-release urea fertilizer from ethylcellulose and superabsorbent coated formulations. Chem. Eng. J..

[380] Alam M.N., Christopher L.P. (2018). Natural cellulose-chitosan cross-linked superabsorbent hydrogels with superior swelling properties. ACS Sustain. Chem. Eng..

[381] Dai H., Huang H. (2017). Enhanced swelling and responsive properties of pineapple peel arboxymethyl cellulose-g-poly (acrylic acid-co-acrylamide) superabsorbent hydrogel by the introduction of carclazyte. J. Agric. Food Chem..

[382] Youssef G., El-Etr W., Zein El-abdeen H., El-Farghal W. (2019). Evaluation of some synthetic soil conditioners and nitrogen rates on nitrogen use efficiency by maize-wheat crops system in calcareous soil. J. Soil Sci. Agric. Eng..

[383] Ni N., Dumont M.-J. (2017). Protein-based hydrogels derived from industrial byproducts containing collagen, keratin, zein and soy. Waste Biomass Valorization.

[384] Nnadi F., Brave C. (2011). Environmentally friendly superabsorbent polymers for water conservation in agricultural lands. J. Soil Environ. Manag..

[385] Ebrahimi Moghadam H., Taghvaei M., Sadeghi H., Zarei M. (2019). Effect of organic coats with superabsorbent polymers on improving the germination and early vigor Milk thistle (Silybum marianum L.) seeds under salinity stress. Desert.

[386] Feng D., Bai B., Wang H., Suo Y. (2017). Novel fabrication of biodegradable superabsorbent microspheres with diffusion barrier through thermo-chemical modification and their potential agriculture applications for water holding and sustained release of fertilizer. J. Agric. Food Chem..

[387] Gómez-Martínez D., Partal P., Martínez I., Gallegos C. (2013). Gluten-based bioplastics with modified controlled-release and hydrophilic properties. Ind. Crops Prod..

[388] Jiménez-Rosado M., Pérez-Puyana V., Cordobés F., Romero A., Guerrero A. (2018). Development of soy protein-based matrices containing zinc as micronutrient for horticulture. Ind. Crops Prod..

[389] McCabe K.G., Currey C.J., Schrader J.A., Grewell D., Behrens J., Graves W.R. (2016). Pelletized soy-based bioplastic fertilizers for container-crop production. HortScience.

[390] Taylor J., Anyango J.O., Taylor J.R.N. (2013). Developments in the science of Zein, Kafirin, and gluten protein bioplastic materials. Cereal Chem..

[391] Ma P.X. (2004). Scaffolds for tissue fabrication. Mater. Today.

[392] Carletti E., Motta A., Migliaresi C., Haycock J.W. (2011). Scaffolds for tissue engineering and 3D cell culture. 3D Cell Culture: Methods and Protocols.

[393] Hollister S.J. (2009). Scaffold design and manufacturing: From concept to clinic. Adv. Mater..

[394] Silva N.H.C.S., Vilela C., Marrucho I.M., Freire C.S.R., Pascoal Neto C., Silvestre A.J.D. (2014). Protein-based materials: From sources to innovative sustainable materials for biomedical applications. J. Mater. Chem. B.

[395] Hu X., Cebe P., Weiss A.S., Omenetto F., Kaplan D.L. (2012). Protein-based composite materials. Mater. Today.

[396] Abou Neel E.A., Bozec L., Knowles J.C., Syed O., Mudera V., Day R., Hyun J.K. (2013). Collagen–Emerging collagen based therapies hit the patient. Adv. Drug Deliv. Rev..

[397] Perez-Puyana V., Romero A., Guerrero A. (2016). Influence of collagen concentration and glutaraldehyde on collagen-based scaffold properties. J. Biomed. Mater. Res. Part A.

[398] Perez-Puyana V., Felix M., Romero A., Guerrero A. (2019). Influence of the processing variables on the microstructure and properties of gelatin-based scaffolds by freeze-drying. J. Appl. Polym. Sci..

[399] Perez-Puyana V., Felix M., Cabrera L., Romero A., Guerrero A. (2019). Development of gelatin/chitosan membranes with controlled microstructure by electrospinning. Iran. Polym. J..

[400] Katoh K., Shibayama M., Tanabe T., Yamauchi K. (2004). Preparation and properties of keratin–poly (vinyl alcohol) blend fiber. J. Appl. Polym. Sci..

[401] Li J., Liu X., Zhang J., Zhang Y., Han Y., Hu J., Li Y. (2012). Synthesis and characterization of wool keratin/hydroxyapatite nanocomposite. J. Biomed. Mater. Res. Part B Appl. Biomater..

[402] Balaji S., Kumar R., Sripriya R., Kakkar P., Ramesh D.V., Reddy P.N.K., Sehgal P.K. (2012). Preparation and comparative characterization of keratin–chitosan and keratin–gelatin composite scaffolds for tissue engineering applications. Mater. Sci. Eng. C.

[403] Jahangirian H., Azizi S., Rafiee-Moghaddam R., Baratvand B., Webster T.J. (2019). Status of plant protein-based green scaffolds for regenerative medicine applications. Biomolecules.

[404] Ozaltin K., Vargun E., Di Martino A., Capakova Z., Lehocky M., Humpolicek P., Kazantseva N., Saha P. (2020). Cell response to PLA scaffolds functionalized with various seaweed polysaccharides. Int. J. Polym. Mater. Polym. Biomater..

[405] Madub K., Goonoo N., Gimié F., Ait Arsa I., Schönherr H., Bhaw-Luximon A. (2021). Green seaweeds ulvan-cellulose scaffolds enhance in vitro cell growth and in vivo angiogenesis for skin tissue engineering. Carbohydr. Polym..

[406] Sasaki K., Ishihara J., Ishihara A., Miura R., Mansurov A., Fukunaga K., Hubbell J.A. (2019). Engineered collagen-binding serum albumin as a drug conjugate carrier for cancer therapy. Sci. Adv..

[407] Reichl S. (2009). Films based on human hair keratin as substrates for cell culture and tissue engineering. Biomaterials.

[408] Jagadeesh D., Kanny K., Prashantha K. (2017). A review on research and development of green composites from plant protein-based polymers. Polym. Compos..

[409] Tian H., Zhou H., Fu H., Li X., Gong W. (2020). Enhanced electrical and dielectric properties of plasticized soy protein bioplastics through incorporation of nanosized carbon black. Polym. Compos..

