# Correction: Álvarez-Castillo et al. Proteins from Agri-Food Industrial Biowastes or Co-Products and Their Applications as Green Materials. *Foods* 2021, *10*, 981

**DOI:** 10.3390/foods14071249

**Published:** 2025-04-03

**Authors:** Estefanía Álvarez-Castillo, Manuel Felix, Carlos Bengoechea, Antonio Guerrero

**Affiliations:** Departamento de Ingeniería Química, Escuela Politécnica Superior, 41011 Sevilla, Spain; malvarez43@us.es (E.Á.-C.); mfelix@us.es (M.F.); aguerrero@us.es (A.G.)

In the original publication [1], ref [379] was cited in error. With this correction, the original reference [379] has been removed, and the citation has been updated to [378]. The related text in Section 5.3, the thirteenth sentence, should now read as follows:

“Moreover, plant nutrients may also be entrapped within superabsorbent matrices for their controlled release, hindering the water losses due to evaporation, and reducing the irrigation [378]”.

The authors state that the scientific conclusions are unaffected. This correction was approved by the Academic Editor. The original publication has also been updated.
